# Linguistic Z-number weighted averaging operators and their application to portfolio selection problem

**DOI:** 10.1371/journal.pone.0227307

**Published:** 2020-01-23

**Authors:** Amir Hosein Mahmoodi, Seyed Jafar Sadjadi, Soheil Sadi-Nezhad, Roya Soltani, Farzad Movahedi Sobhani

**Affiliations:** 1 Department of Industrial Engineering, Science and Research branch, Islamic Azad University, Tehran, Iran; 2 Department of Industrial Engineering, Iran University of Science and Technology, Narmak, Tehran, Iran; 3 Department of Statistic and Actuarial Science, University of Waterloo, Ontario, Canada; The Bucharest University of Economic Studies, ROMANIA

## Abstract

Z-numbers can generate a more flexible structure to model the real information because of capturing expert’s reliability. Moreover, various semantics can flexibly be reflected by linguistic terms under various circumstances. Thus, this study aims to model the portfolio selection problems based on aggregation operators under linguistic Z-number environment. Therefore, a multi-stage methodology is proposed and linguistic Z-numbers are applied to describe the assessment information. Moreover, the weighted averaging (WA) aggregation operator, the ordered weighted averaging (OWA) aggregation operator and the hybrid weighted averaging (HWA) aggregation operator are developed to fuse the input arguments under the linguistic Z-number environment. Then, using the max-score rule and the score-accuracy trade-off rule, three qualitative portfolio models are presented to allocate the optimal assets. These models are suitable for general investors and risky investors. Finally, to illustrate the validity of the proposed qualitative approach, a real case including 20 corporations of Tehran stock exchange market in Iran is provided and the obtained results are analyzed. The results show that combining linguistic Z-numbers with portfolio selection processes can increase the tendencies and capabilities of investors in the capital market and it helps them manage their portfolios efficiently.

## 1. Introduction

In the real world, decisions are usually made based on information, which is uncertain, incomplete and vague. Moreover, with increasing complexity in the decision-making environment, Decision-Makers (DMs) no longer are satisfied with the classic or deterministic techniques for modeling their knowledge about events and they are intending to make more fruitful decisions using more general data. The fuzzy set theory, which was introduced by Zhdeh [1], has created massive progress in the representation of uncertain and ambiguous data and has successfully been applied in the different area. Following Zadeh [1], some authors developed the fuzzy set theory and presented its extended forms such as the interval-valued fuzzy sets [2], the type-2 fuzzy sets [3], the fuzzy multi-sets [4], the intuitionistic fuzzy sets [5], the hesitant fuzzy sets [6], the interval-valued hesitant fuzzy sets [7], picture fuzzy sets [8], Picture Hesitant Fuzzy Set [9], Proportional hesitant fuzzy linguistic term set [10] and proportional interval type-2 hesitant fuzzy set [11]. However, the fuzzy set theory and its extended forms are inefficient at representing the reliability of the information. Hence, to fill this limitation, Zadeh [12] introduced a new concept called Z-number and created a more general structure for modeling uncertain information related to the real-world phenomena. In the literature, the concept of Z-numbers has been investigated into two fields. The first field is the basic studies such as conversion techniques [13, 14], arithmetic operations over Z-numbers [15–17], ranking methods [18–22] and development researches [23–26]. The second field is related to the application of Z-numbers in decision-making and optimization problems. Some authors (such as [27–31]) used the concept of Z-numbers in decision-making modeling and presented different decision-making methods under Z-number environment. The prominent feature of Z-numbers is that they reflect the reliability of information in addition to uncertainty and ambiguity simultaneously. Moreover, Z-numbers can combine possibilistic and probabilistic restrictions simultaneously.

Often, experts express their opinions and assessment information by using linguistic terms because of fuzziness, uncertainty and ambiguity available in their knowledge. For example, to assess the financial performance of a firm or estimate the future return of an asset, experts can utilize linguistic terms such as “low”, “medium”, and “high”. An important advantage of linguistic terms is that they can enhance the credibility and flexibility of the decision-making models [32]. Therefore, linguistic terms were successfully used in various areas [33–38]. On the other hand, the fuzziness and randomness existing in linguistic terms accurately match up the constraint and the reliability measure of Z-numbers [25]. Hence, Wang et al. [25] incorporated linguistic terms with the concept of Z-numbers and introduced linguistic Z-numbers and extended their operations. Also, Peng and Wang [26] introduced hesitant uncertain linguistic Z-numbers (HULZNs). The prominent feature of the linguistic Z-numbers (LZNs) is that they capture both the fuzziness and randomness related to qualitative data simultaneously. For instance, the financial performance of a firm or the future return of an asset can be evaluated by using a linguistic Z-number so that linguistic terms such as “low”, “medium” and “high” can be applied to describe fuzzy constraint and linguistic terms such as “seldom”, “sometimes” and “usually” can be used to represent the probability measure. Hence, the financial performance of a firm can be assessed by using a linguistic Z-number such as (high, usually). As discussed, not only does a linguistic Z-number characterize the uncertainty of a variable by linguistic terms, but also it determines the reliability of this linguistic term by another linguistic term. This advantage causes the concept of linguistic Z-numbers to be beneficial in financial markets. Therefore, in this study, the linguistic Z-numbers are applied to better represent the qualitative financial data that expert determines.

One of the most important methods for fusing assessment information under various type of uncertain environment is aggregation operator. The techniques based on aggregation operators are premiere in comparison with traditional decision-making methods because they can obtain comprehensive values [39]. Therefore, many authors (such as [38–48]) developed different kinds of aggregation operators under deterministic and uncertain environments. However, there are only two aggregation operators to aggregate qualitative data under hesitant linguistic Z-number environment including the hesitant uncertain linguistic Z-number power weighted average operator and the hesitant uncertain linguistic Z-number power weighted geometric operator [26]. Hence, in this paper, three of the most common aggregation operators, including weighted averaging (WA) operator, ordered weighted averaging (OWA) operator and hybrid weighted averaging (HWA) operator are developed under Z-number linguistic environment. Moreover, three new aggregation operators naming linguistic Z-number weighted averaging (LZWA) operator, linguistic Z-number ordered weighted averaging (LZOWA) operator, and linguistic Z-number hybrid weighted averaging (LZHWA) operator are introduced.

One of the most important problems in investment process is to choose the optimal collection of assets in a portfolio. Portfolio selection problem is modeled to attain one of the goals of investment management which is selecting a proper portfolio for investors. In the financial markets, investors usually face situations in which the financial goals such as return and risk have conflict together. Hence, Markowitz [49] presented a quantitative model called modern portfolio theory to select the optimal portfolio based on a trade-off between the expected return and the risk. According to Markowitz’s model, many authors developed his mean-variance model by supposing the return of assets as a random variable. Hence, only historical information was applied to estimate the future return of assets. However, there are many non-random factors in the financial market that probability theory is unable to resolve. Therefore, to overcome this limitation, many scholars used the fuzzy set theory to model the portfolio selection problem. Although these scholars discerned the fuzziness in the financial markets, they ignored the reliability of the information in their researches. Z-numbers is a concept to consider the reliability of information which leads to rational selections when encountering different data. A Z-number is an ordered pair of fuzzy numbers. The first fuzzy component makes a restriction on the uncertain variable and the second component describes the reliability of the first component. Consequently, a Z-number both characterizes the uncertainty of a variable using a fuzzy number and displays the reliability of this fuzzy number by using another fuzzy number. This property causes Z-numbers more suitable in financial markets. Therefore, in this study, some linguistic Z-number aggregation operators are developed and a multi-stage approach is proposed to handle the qualitative portfolio selection problem under linguistic Z-number environment. The main aims of this paper are briefly highlighted as follows:

1. linguistic Z-numbers are applied to describe the assessment data relevant to the financial performances of corporations. This feature makes the proposed approach and the proposed models more flexible in comparison with the traditional portfolio models.
2. Weighted averaging (WA) operator, ordered weighted averaging (OWA) operator and hybrid weighted averaging (HWA) operator under linguistic Z-number environment are developed. Moreover, linguistic Z-number weighted averaging (LZWA) operator, linguistic Z-number ordered weighted averaging (LZOWA) operator and linguistic Z-number hybrid weighted averaging (LZHWA) operator are introduced.
3. A multi-stage approach for modeling the qualitative portfolio problem is proposed by applying LZWA, LZOWA, and LZHWA operators under linguistic Z-number environment. Moreover, three qualitative portfolio models are presented. The proposed models consider investors’ preferences to construct more diversified portfolios and are suitable for both general investors and risky investors.

The rest of this study is arranged as follows: Section 2 includes the necessary prerequisite definitions. In Section 3, we introduce some aggregation operators under linguistic Z-number environment and present their properties. Three new qualitative portfolio models are developed in Section 4. Section 5 provides the required actual data as a case study and the obtained results and the sensitivity analysis are shown. Finally, concluding remarks and future work suggestions are presented in Section 6.

## 2. Preliminaries

### 2.1. Linguistic terms set and linguistic scale functions (LSFs)

Suppose *s*_*l*_∈*S* be a conceivable value of linguistic variable, where *S* = {*s*_*l*_|*l* = 0,1,…,2*m*}. *S* should include the following properties [50, 51]:

- *S* is ordered: *s*_*l*_<*s*_*k*_ *if and only if l*<*k*.
- *S* conforms negation operator: *neg*(*s*_*l*_) = *s*_2*m*−*l*_

It is clear that T is a discrete linguistic term set. Because the computational results do not usually match the members of T, Xu [52–54] has introduced a continuous linguistic term set $\bar{S}$, where $\bar{S}=\{s_{i}|i\in [0,l]\}$, to prevent the loss of the obtained information.

Since Computation with linguistic terms (LTs) is placed within the category of computing with words (CWW), carrying out the arithmetic operation with them is not easy. Hence, some functions called linguistic scale functions (LSFs) have been defined to simplify the computation under linguistic environment [38, 53]. Different semantics are devoted to linguistic terms by using LSFs under various circumstances to apply information more flexible. There is a strictly monotonically ascending connection among every *s*_*l*_∈*T* and its label (*l*) [38, 53].

Definition 1. Let *S* = {*s*_*l*_|*l* = 0,1,…,2*m*} be a set of discrete linguistic terms with odd cardinality. The linguistic scale function f is defined as follows [38]:

$$f:\;s_{l}\to \theta_{l}\;(l=0,1,\ldots ,2m)$$

Where *θ*_*l*_ is a positive real number and 0≤*θ*_0_≤*θ*_1_≤⋯≤*θ*_2*m*_. *θ*_*l*_ characterizes the priorities of DMs when *s*_*l*_∈*S* is chosen to describe their opinions.

Some LSFs are introduced as follows [25, 38]:

$$f_{1}(s_{l})=\theta_{l}=\frac{l}{2m}\;(l=0,1,\ldots ,2m)\;and\;\theta_{l}\epsilon [0,1]$$ (1)

$$f_{2}(s_{l})=\theta_{l}={\left(\frac{l}{2m}\right)}^{m}\;(l=0,1,\ldots ,2m)$$ (2)

$$f_{3}(s_{l})=\theta_{l}={\left(\frac{l}{2m}\right)}^{\frac{1}{m}}\;(l=0,1,\ldots ,2m)$$ (3)

$$f_{4}(s_{l})=\theta_{l}=\left\{ \begin{array}{c} \frac{m^{\alpha }-{\left(m-l\right)}^{\alpha }}{2m^{\alpha }}\;(l=0,1,\ldots ,m)\\ \frac{m^{\beta }+{\left(l-m\right)}^{\beta }}{2m^{\beta }}\;(l=m+1,\ldots ,2m) \end{array}\right.$$ (4)

Where *α and β*∈(0,1].

### 2.2. Z-numbers and linguistic Z-numbers

This subsection covers the definition of Z-numbers, uncertain linguistic Z-numbers, and their operation.

#### 2.2.1. Z-number

Uncertainty is an inseparable feature of the real problems. Always, DMs use their uncertain data, knowledge, and experiments to select the best solutions. In order to make more beneficial decisions, this information must be reliable. Hence, Zadeh [12] introduced the concept of Z-numbers to better represent uncertain data with the incorporation of partial reliability and fussiness.

**Definition 2.** [12] (Z-number). An ordered pair of fuzzy numbers as $\left(\tilde{A},\tilde{B}\right)$ shows a Z-number. Such that the values, which can be assigned to an uncertain variable X, are represented by using the first component as a fuzzy constraint, and a soft restriction on partial reliability of the first component is determined by the second component, $\tilde{B}$. Often $\tilde{A}$ and $\tilde{B}$ are expressed by using linguistic terms.

#### 2.2.2. Linguistic Z-numbers

Following to Zadeh [12], two forms of Z-numbers have been developed to better describe uncertain data with consideration of partial reliability. Peng and Wang [26] introduced hesitant uncertain linguistic Z-numbers by using the concept of Z-numbers and linguistic terms. Wang et al. [25] extended a new form of Z-numbers called linguistic Z-numbers in order to measure the reliability of the real phenomena and describe the qualitative data, simultaneously.

**Definition 3.** [25] (Linguistic Z-numbers). Consider a universe of discourse U. Let two finite discrete linguistic term sets representing different preference data are defined as $S=\{s_{0},s_{1},\ldots ,s_{2m}\}\;and\;T^{\prime }=\{s_{0}^{\prime },s_{1}^{\prime },\ldots ,s_{2n}^{\prime }\}$ where m and n are nonnegative integers. Therefore, a linguistic Z-number set in U is defined as follows:

$$Z=\{(u,A_{\emptyset (u)},B_{\varphi (u)})|u\epsilon U\}$$ (5)

Where *A*_∅(*u*)_ is a fuzzy constraint on the values which can be assigned to the uncertain variable and *B*_*φ*(*u*)_ characterizes a reliability measure of the first component. *A*_∅(*u*)_ and *B*_*φ*(*u*)_ are described by using uncertain linguistic terms.

#### 2.2.3. The arithmetic operations over uncertain linguistic Z-numbers

Wang et al. [25] developed some arithmetic operations for LZNs. The proposed operations maintain both the flexibility of linguistic term sets and the reliability value of Z-numbers.

**Definition 4.** [25] Suppose two linguistic Z-numbers are defined as *z*_*i*_ = (*A*_∅(*i*)_, *B*_*φ*(*i*)_) and *z*_*j*_ = (*A*_∅(*j*)_, *B*_*φ*(*j*)_). f* and g* functions can be selected from between *f*_1_(*s*_*l*_), *f*_2_(*s*_*l*_), *f*_3_(*s*_*l*_) and *f*_4_(*s*_*l*_). Hence, some operations under linguistic Z-number environment are defined as follows:

$$neg(z_{i})=\left(f^{*-1}(f^{*}(A_{2m})-f^{*}(A_{\emptyset (i)})),g^{*-1}(g^{*}(B_{2n})-g^{*}(B_{\varphi (i)}))\right)$$ (6)

$$z_{i}+z_{j}=\left(f^{*-1}(f^{*}(A_{\emptyset (i)})+f^{*}(A_{\emptyset (j)})),g^{*-1}(\frac{f^{*}(A_{\emptyset (i)})\times g^{*}(B_{\varphi (i)})+f^{*}(A_{\emptyset (j)})\times g^{*}(B_{\varphi (j)})}{f^{*}(A_{\emptyset (i)})+f^{*}(A_{\emptyset (j)})})\right)$$ (7)

$$\rho z_{i}=(f^{*-1}(\rho f^{*}(A_{\emptyset (i)})),B_{\varphi (i)})\;\rho \geq 0$$ (8)

$$z_{i}\times z_{j}=\left(f^{*-1}(f^{*}(A_{\emptyset (i)})f^{*}(A_{\emptyset (j)})),g^{*-1}(g^{*}(B_{\varphi (i)})g^{*}(B_{\varphi (j)}))\right)$$ (9)

$$z_{i}^{\rho }=\left(f^{*-1}(f^{*}{\left(A_{\emptyset (i)}\right)}^{\rho }),g^{*-1}(g^{*}{\left(B_{\varphi (i)}\right)}^{\rho })\right)\;\rho \geq 0$$ (10)

**Definition 5.** [25] Suppose *z*_*i*_ = (*A*_∅(*i*)_, *B*_*φ*(*i*)_) be a linguistic Z-numbers. Then, the score function of linguistic Z-number is equal to:

$$S(z_{i})=f^{*}\left(A_{\emptyset (i)}\right)\times g^{*}\left(B_{\varphi (i)}\right)$$ (11)

The accuracy function of linguistic Z-number is as follows:

$$A(z_{i})=f^{*}(A_{\emptyset (i)})\times \left(1-g^{*}(B_{\varphi (i)})\right)$$ (12)

Using the score and accuracy functions, a comparison technique is defined for two LZNs as follows (Wang et al., 2017):

- If *A*_∅(*i*)_>*A*_∅(*j*)_ and *B*_*φ*(*i*)_>*B*_*φ*(*j*)_, then *z*_*i*_>*z*_*j*_
- If *S*(*z*_*i*_)>*S*(*z*_*j*_), then *z*_*i*_>*z*_*j*_
- If *S*(*z*_*i*_) = *S*(*z*_*j*_), then

If *A*(*z*_*i*_)>*A*(*z*_*j*_), then *z*_*i*_>*z*_*j*_

If *A*(*z*_*i*_)<*A*(*z*_*j*_), then *z*_*i*_<*z*_*j*_

### 2.3. Aggregation operator

**Definition 6.** [55] A weighted averaging (WA) operator is mapping *WA*: *R*^*n*^→*R* which has an associated vector *W* = (*w*_1_,*w*_2_,…,*w*_*n*_) such that *w*_*i*_∈[0,1] and $\sum_{i=1}^{n}w_{i}=1$. Then, the weighted averaging (WA) operator is defined as follows:

$${WA}_{w}(a_{1},a_{2},\ldots ,a_{n})=\sum_{i=1}^{n}w_{i}a_{i}$$ (13)

**Definition 7.** [56] An ordered weighted averaging (OWA) operator is mapping *OWA*: *R*^*n*^→*R* which has an associated vector *W* = (*w*_1_,*w*_2_,…,*w*_*n*_) such that *w*_*i*_∈[0,1] and $\sum_{i=1}^{n}w_{i}=1$. Then, the ordered weighted averaging (OWA) operator is defined as follows:

$${OWA}_{w}(a_{1},a_{2},\ldots ,a_{n})=\sum_{i=1}^{n}w_{i}b_{i}$$ (14)

where *b*_*i*_ is the *i*^*th*^ largest of *a*_*i*_ (*i* = 1,…,*n*).

**Definition 8.** [57] An hybrid weighted averaging (HWA) operator is mapping *HWA*: *R*^*n*^→*R*, and it has an associated vector *W* = (*w*_1_,*w*_2_,…,*w*_*n*_) such that *w*_*i*_∈[0,1] and $\sum_{i=1}^{n}w_{i}=1$. Then, the hybrid weighted averaging (HWA) operator is defined as follows:

$${HWA}_{w,\omega }(a_{1},a_{2},\ldots ,a_{n})=\sum_{i=1}^{n}w_{i}{\dot{a}}_{\sigma (i)}$$ (15)

Where ${\dot{a}}_{\sigma (i)}$ is the *i*^*th*^ largest of ${\dot{a}}_{i}(i=1,\ldots ,n)$, ${\dot{a}}_{i}=n\omega a_{i}$ and *ω* = (*ω*_1_,*ω*_2_,…,*ω*_*n*_) be the weighted vector of *a*_*i*_ such that *ω*_*i*_∈[0,1] and $\sum_{i=1}^{n}\omega_{i}=1$. *n* is the balancing coefficient.

The mentioned aggregation operators have typically been applied to fuse the exact input arguments. Hence, some authors (such as [38, 43, 54, 57, 58]) developed these aggregation operators under different kinds of fuzzy environments. However, they ignored the reliability of data in their aggregation operations. To overcome this deficiency, these mentioned aggregation operators will be extended under linguistic Z-number environment in the next section.

## 3. Linguistic Z-number aggregation operators

This section covers three aggregation operators under linguistic Z-number environment. The description of linguistic Z-number aggregation operators and its related properties and theorems are indicated in the following.

### 3.1. Linguistic Z-number weighted averaging (LZWA) aggregation operator

**Definition 9.** Suppose *Z* = {*z*_*i*_ = (*A*_∅(*i*)_,*B*_*φ*(*i*)_)|*i* = 1,…,*n*} be a set of linguistic Z-numbers (LZNs) and *W* = (*w*_1_,*w*_2_,…,*w*_*n*_) is the weight vector of *z*_*i*_ (*i* = 1,…,*n*) such that *w*_*i*_∈[0,1] and $\sum_{i=1}^{n}w_{i}=1$. Then, linguistic Z-number weighted averaging (LZWA) aggregation operator can be defined as follows:

$${LZWA}_{w}(z_{1},z_{2},\ldots ,z_{n})=\sum_{i=1}^{n}w_{i}z_{i}$$ (16)

Based on the operations of linguistic Z-numbers represented in Definition 4, we can get the following result.

**Theorem 1.** Let *z*_*i*_ = (*A*_∅(*i*)_,*B*_*φ*(*i*)_) (*i* = 1,…,*n*) be a set of LZNs and *W* = (*w*_1_,*w*_2_,…,*w*_*n*_) is the weight vector of *z*_*i*_ (*i* = 1,…,*n*) such that *w*_*i*_∈[0,1] and $\sum_{i=1}^{n}w_{i}=1$. Then, the aggregated result acquired based on LZWA operator is also a LZN and is indicated as follows:

$${LZWA}_{w}(z_{1},z_{2},\ldots ,z_{n})=\left(f^{{*}^{-1}}(\sum_{i=1}^{n}w_{i}f^{*}(A_{\emptyset (i)})),g^{{*}^{-1}}(\frac{\sum_{i=1}^{n}\left(w_{i}f^{*}(A_{\emptyset (i)})\times g^{*}(B_{\varphi (i)})\right)}{\sum_{i=1}^{n}w_{i}f^{*}(A_{\emptyset (i)})})\right)$$ (17)

**Proof**. According to Definition 4, it is clear that the aggregated value is also a LZN. Now, by applying the mathematical induction method, Eq (17) is easily proved in the following.

Firstly, it is assumed *n* = 2. Then, according to Definition 4, we have:

$$w_{1}z_{1}=\left(f^{{*}^{-1}}(w_{1}f^{*}(A_{\emptyset (1)})),B_{\varphi (1)}\right)$$

$$w_{2}z_{2}=\left(f^{{*}^{-1}}(w_{2}f^{*}(A_{\emptyset (2)})),B_{\varphi (2)}\right)$$

$${LZWA}_{w}(z_{1},z_{2})=\left(f^{{*}^{-1}}(\left(w_{1}f^{*}(A_{\emptyset (1)}))+(w_{2}f^{*}(A_{\emptyset (2)})\right)),g^{{*}^{-1}}(\frac{\left(\left(w_{1}f^{*}(A_{\emptyset (1)}))\times g^{*}(B_{\varphi (1)}\right))+(\left(w_{2}f^{*}(A_{\emptyset (2)}))\times g^{*}(B_{\varphi (2)}\right)\right)}{\left(\left(w_{1}f^{*}(A_{\emptyset (1)}))+(w_{2}f^{*}(A_{\emptyset (2)})\right)\right)})\right)$$

Clearly, Theorem 1 is true for the case of *n* = 2. Now, it is assumed that this theorem is true for *n* = *k*, then we have:

$${LZWA}_{w}(z_{1},z_{2},\ldots ,z_{k})=\left(f^{{*}^{-1}}(\sum_{i=1}^{k}w_{i}f^{*}(A_{\emptyset (i)})),g^{{*}^{-1}}(\frac{\sum_{i=1}^{k}\left(w_{i}f^{*}(A_{\emptyset (i)})\times g^{*}(B_{\varphi (i)})\right)}{\sum_{i=1}^{k}w_{i}f^{*}(A_{\emptyset (i)})})\right)$$

Finally, for the case of *n* = *k*+1, we can obtain the following expression:

$${LZWA}_{w}(z_{1},z_{2},\ldots ,z_{k+1})=\left(f^{{*}^{-1}}\left(\sum_{i=1}^{k+1}w_{i}f^{*}(A_{\emptyset (i)})\right),g^{{*}^{-1}}(\frac{\sum_{i=1}^{k+1}\left(w_{i}f^{*}(A_{\emptyset (i)})\times g^{*}(B_{\varphi (i)})\right)}{\sum_{i=1}^{k+1}w_{i}f^{*}(A_{\emptyset (i)})})\right)$$

Since this theorem is true for the case of *n* = *k*, it will be also true for the case of *n* = *k*+1. Consequently, according to the mathematical induction, Eq (17) is true for all *n*. ∎

It is easy to prove that the LZWA operator has the following properties.

**Theorem 2.** (Idempotency). Let *z*_*i*_ = (*A*_∅(*i*)_,*B*_*φ*(*i*)_) (*i* = 1,…,*n*) be a set of LZNs and *W* = (*w*_1_,*w*_2_,…,*w*_*n*_) is the weight vector of *z*_*i*_ (*i* = 1,…,*n*) such that *w*_*i*_∈[0,1] and $\sum_{i=1}^{n}w_{i}=1$. If all *z*_*i*_ are equal, i.e., *z*_*i*_ = *z* = (*A*_∅(.)_,*B*_*φ*(.)_), for all *i*, then:

$${LZWA}_{w}(z_{1},z_{2},\ldots ,z_{n})=z$$

**Proof.**

$${LZWA}_{w}(z_{1},z_{2},\ldots ,z_{n})={LZWA}_{w}(z,z,\ldots ,z)=\left(f^{{*}^{-1}}(\sum_{i=1}^{n}w_{i}f^{*}(A_{\emptyset (.)})),g^{{*}^{-1}}(\frac{\sum_{i=1}^{n}\left(w_{i}f^{*}(A_{\emptyset (.)})\times g^{*}(B_{\varphi (.)})\right)}{\sum_{i=1}^{n}w_{i}f^{*}(A_{\emptyset (.)})})\right)=\left(f^{{*}^{-1}}(f^{*}(A_{\emptyset (.)})\sum_{i=1}^{n}w_{i}),g^{{*}^{-1}}(\frac{(f^{*}(A_{\emptyset (.)})\times g^{*}(B_{\varphi (.)}))\sum_{i=1}^{n}\left(w_{i}\right)}{f^{*}(A_{\emptyset (.)})\sum_{i=1}^{n}w_{i}})\right)=(A_{\emptyset (.)},B_{\varphi (.)})=z∎$$

**Theorem 3.** (Boundedness). Let *z*_*i*_ = (*A*_∅(*i*)_,*B*_*φ*(*i*)_) (*i* = 1,…,*n*) be a set of LZNs and *W* = (*w*_1_,*w*_2_,…,*w*_*n*_) is the weight vector of *z*_*i*_ (*i* = 1,…,*n*) such that *w*_*i*_∈[0,1] and $\sum_{i=1}^{n}w_{i}=1$, and let:

$$z^{-}=\min_{i}z_{i}=(A_{z^{-}},B_{z^{-}})=\left(\min_{i}\left(A_{\emptyset (i)}\right),\min_{i}\left(B_{\varphi (i)}\right)\right)$$

$$z^{+}=\max_{i}z_{i}=(A_{z^{+}},B_{z^{+}})=\left(\max_{i}\left(A_{\emptyset (i)}\right),\max_{i}\left(B_{\varphi (i)}\right)\right)$$

then,

$$z^{-}\leq {LZWA}_{w}(z_{1},z_{2},\ldots ,z_{n})\leq z^{+}$$

Proof. Let *LZWA*_*w*_(*z*_1_,*z*_2_,…,*z*_*n*_) = *z*_*T*_ = (*A*_∅(*T*)_,*B*_*φ*(*T*)_), since $A_{z^{-}}\leq A_{\emptyset (i)}$ and $B_{z^{-}}\leq B_{\varphi (i)}$. Thus, $f^{*}(A_{z^{-}})\leq f^{*}(A_{\emptyset (i)})$ and $g^{*}(B_{z^{-}})\leq g^{*}(B_{\varphi (i)})$ and *z*^−^<*z*_*i*_. Then, we have:

$$S(z^{-})=f^{*}(A_{z^{-}})\times g^{*}(B_{z^{-}})\leq S(z_{T})=f^{*}(A_{\emptyset (T)})\times g^{*}(B_{\varphi (T)})$$

Where $z_{T}=(A_{\emptyset (T)},B_{\varphi (T)})=\left(f^{{*}^{-1}}(\sum_{i=1}^{n}w_{i}f^{*}(A_{\emptyset (i)})),g^{{*}^{-1}}(\frac{\sum_{i=1}^{n}\left(w_{i}f^{*}(A_{\emptyset (i)})\times g^{*}(B_{\varphi (i)})\right)}{\sum_{i=1}^{n}w_{i}f^{*}(A_{\emptyset (i)})})\right)$. Therefore, *z*^−^≤*LZWA*_*w*_(*z*_1_,*z*_2_,…,*z*_*n*_) can be obtained based on the comparison method of linguistic Z-numbers presented in Definition 5. Similarly, *LZWA*_*w*_(*z*_1_,*z*_2_,…,*z*_*n*_)≤*z*^+^ can also be obtained. Thus, *z*^−^≤*LZWA*_*w*_(*z*_1_,*z*_2_,…,*z*_*n*_)≤*z*^+^ ∎.

**Theorem 4.** (Monotonicity). Let *z*_*i*_ = (*A*_∅(*i*)_,*B*_*φ*(*i*)_) (*i* = 1,…,*n*) and $z_{i}^{*}=(A_{\emptyset (i)}^{*},B_{\varphi (i)}^{*})\;(i=1,\ldots ,n)$ be two sets of LZNs and *W* = (*w*_1_,*w*_2_,…,*w*_*n*_) is the weight vector of *z*_*i*_ *and* $z_{i}^{*}$ (*i* = 1,…,*n*) such that *w*_*i*_∈[0,1] and $\sum_{i=1}^{n}w_{i}=1$. If $A_{\emptyset (i)}\leq A_{\emptyset (i)}^{*}$ and $B_{\varphi (i)}\leq B_{\varphi (i)}^{*}$, for all *i*, then,

$${LZWA}_{w}(z_{1},z_{2},\ldots ,z_{n})\leq {LZWA}_{w}(z_{1}^{*},z_{2}^{*},\ldots ,z_{n}^{*})$$

Proof. Since $A_{\emptyset (i)}\leq A_{\emptyset (i)}^{*}$ and $B_{\varphi (i)}\leq B_{\varphi (i)}^{*}$, for all *i*, according to Definition 5, it can be resulted that $z_{i}\leq z_{i}^{*}$, for all *i*. Therefore, $f^{*}(A_{\emptyset (i)})\leq f^{*}(A_{\emptyset (i)}^{*})$ and $g^{*}(B_{\varphi (i)})\leq g^{*}(B_{\varphi (i)}^{*})$. Consequently, we have:

$${LZWA}_{w}(z_{1},z_{2},\ldots ,z_{n})=\left(f^{{*}^{-1}}(\sum_{i=1}^{n}w_{i}f^{*}(A_{\emptyset (i)})),g^{{*}^{-1}}(\frac{\sum_{i=1}^{n}\left(w_{i}f^{*}(A_{\emptyset (i)})\times g^{*}(B_{\varphi (i)})\right)}{\sum_{i=1}^{n}w_{i}f^{*}(A_{\emptyset (i)})})\right)\leq \left(f^{{*}^{-1}}(\sum_{i=1}^{n}w_{i}f^{*}(A_{\emptyset (i)}^{*})),g^{{*}^{-1}}(\frac{\sum_{i=1}^{n}\left(w_{i}f^{*}(A_{\emptyset (i)}^{*})\times g^{*}(B_{\varphi (i)}^{*})\right)}{\sum_{i=1}^{n}w_{i}f^{*}(A_{\emptyset (i)}^{*})})\right)={LZWA}_{w}(z_{1}^{*},z_{2}^{*},\ldots ,z_{n}^{*})\;∎$$

**Theorem 5.** Let *z*_*i*_ = (*A*_∅(*i*)_,*B*_*φ*(*i*)_) (*i* = 1,…,*n*) be a set of LZNs and *W* = (*w*_1_,*w*_2_,…,*w*_*n*_) is the weight vector of *z*_*i*_ (*i* = 1,…,*n*) such that *w*_*i*_∈[0,1] and $\sum_{i=1}^{n}w_{i}=1$. For each *q*>0, we can indicate:

$${LZWA}_{w}(qz_{1},qz_{2},\ldots ,qz_{n})={qLZWA}_{w}(z_{1},z_{2},\ldots ,z_{n})$$

**Proof.**

$${LZWA}_{w}(qz_{1},qz_{2},\ldots ,qz_{n})=\left(f^{{*}^{-1}}\left(\sum_{i=1}^{n}{qw}_{i}f^{*}(A_{\emptyset (i)})\right),g^{{*}^{-1}}(\frac{\sum_{i=1}^{n}\left(w_{i}f^{*}(A_{\emptyset (i)})\times g^{*}(B_{\varphi (i)})\right)}{\sum_{i=1}^{n}w_{i}f^{*}(A_{\emptyset (i)})})\right)=\left(f^{{*}^{-1}}\left(q\sum_{i=1}^{n}w_{i}f^{*}(A_{\emptyset (i)})\right),g^{{*}^{-1}}(\frac{\sum_{i=1}^{n}\left(w_{i}f^{*}(A_{\emptyset (i)})\times g^{*}(B_{\varphi (i)})\right)}{\sum_{i=1}^{n}w_{i}f^{*}(A_{\emptyset (i)})})\right)$$

$${qLZWA}_{w}(z_{1},z_{2},\ldots ,z_{n})=q\left(f^{{*}^{-1}}\left(\sum_{i=1}^{n}w_{i}f^{*}(A_{\emptyset (i)})\right),g^{{*}^{-1}}(\frac{\sum_{i=1}^{n}\left(w_{i}f^{*}(A_{\emptyset (i)})\times g^{*}(B_{\varphi (i)})\right)}{\sum_{i=1}^{n}w_{i}f^{*}(A_{\emptyset (i)})})\right)=\left(f^{{*}^{-1}}(q\sum_{i=1}^{n}w_{i}f^{*}(A_{\emptyset (i)})),g^{{*}^{-1}}(\frac{\sum_{i=1}^{n}\left(w_{i}f^{*}(A_{\emptyset (i)})\times g^{*}(B_{\varphi (i)})\right)}{\sum_{i=1}^{n}w_{i}f^{*}(A_{\emptyset (i)})})\right)$$

Thus

$${LZWA}_{w}(qz_{1},qz_{2},\ldots ,qz_{n})={qLZWA}_{w}(z_{1},z_{2},\ldots ,z_{n})\;∎$$

It should be noted that the LZWA operator does not possess commutativity. For example, let $z_{1}=(s_{6},s_{3}^{\prime }),\;z_{2}=(s_{5},s_{4}^{\prime }),\;z_{3}=(s_{4},s_{3}^{\prime })$ and $z_{4}=(s_{3},s_{2}^{\prime })$, then ${LZWA}_{w}(z_{1},z_{2},z_{3},z_{4})=(s_{4.5},s_{3.28}^{\prime })$ and ${LZWA}_{w}(z_{4},z_{1},z_{3},z_{2})=(s_{4.69},s_{3.06}^{\prime })$ for *w* = (0.155,0.345,0.345,0.155).

In the following, the special case of LZWA operator is presented:

If $W=(\frac{1}{n},\frac{1}{n},\ldots ,\frac{1}{n})$, then the LZWA operator is reduced to linguistic Z-number averaging (LZA) operator, which is defined as follows:

$${LZWA}_{w}(z_{1},z_{2},\ldots ,z_{n})=\frac{1}{n}\sum_{i=1}^{n}z_{i}$$

### 3.2. Linguistic Z-number ordered weighted averaging (LZOWA) aggregation operator

**Definition 10.** Suppose *Z* = {*z*_*i*_ = (*A*_∅(*i*)_,*B*_*φ*(*i*)_)|*i* = 1,…,*n*} be a set of linguistic Z-numbers (LZNs) and *W* = (*w*_1_,*w*_2_,…,*w*_*n*_) is the weight vector of *z*_*i*_ (*i* = 1,…,*n*) such that *w*_*i*_∈[0,1] and $\sum_{i=1}^{n}w_{i}=1$. Then, linguistic Z-number ordered weighted averaging (LZOWA) aggregation operator can be defined as follows:

$${LZOWA}_{w}(z_{1},z_{2},\ldots ,z_{n})=\sum_{i=1}^{n}w_{i}z_{\sigma (i)}$$ (18)

Where (*σ*(1),*σ*(2),…,*σ*(*n*)) is a permutation of (1,…,*n*) such that *z*_*σ*(*i*−1)_≥*z*_*σ*(*i*)_.

Based on the operations of linguistic Z-numbers represented in Definition 4, we can get the following result.

**Theorem 6.** Let *z*_*i*_ = (*A*_∅(*i*)_,*B*_*φ*(*i*)_) (*i* = 1,…,*n*) be a set of LZNs and *W* = (*w*_1_,*w*_2_,…,*w*_*n*_) is the weight vector of *z*_*i*_ (*i* = 1,…,*n*) such that *w*_*i*_∈[0,1] and $\sum_{i=1}^{n}w_{i}=1$. Then, the aggregated result acquired based on LZOWA operator is also a LZN and is indicated as follows:

$${LZOWA}_{w}(z_{1},z_{2},\ldots ,z_{n})=\left(f^{{*}^{-1}}\left(\sum_{i=1}^{n}w_{i}f^{*}(A_{\emptyset (\sigma (i))})\right),g^{{*}^{-1}}(\frac{\sum_{i=1}^{n}\left(w_{i}f^{*}(A_{\emptyset (\sigma (i))})\times g^{*}(B_{\varphi (\sigma (i))})\right)}{\sum_{i=1}^{n}w_{i}f^{*}(A_{\emptyset (\sigma (i))})})\right)$$ (19)

Similarly, Theorem 6 can easily be proven by using Definition 5 and the mathematical induction technique.

It is easy to prove that the LZOWA operator has the following properties.

**Theorem 7.** (Idempotency). Let *z*_*i*_ = (*A*_∅(*i*)_,*B*_*φ*(*i*)_) (*i* = 1,…,*n*) be a set of LZNs and *W* = (*w*_1_,*w*_2_,…,*w*_*n*_) is the weight vector of *z*_*i*_ (*i* = 1,…,*n*) such that *w*_*i*_∈[0,1] and $\sum_{i=1}^{n}w_{i}=1$. If all *z*_*i*_ are equal, i.e., *z*_*i*_ = *z* = (*A*_∅(.)_,*B*_*φ*(.)_) for all *i*, then:

$${LZOWA}_{w}(z_{1},z_{2},\ldots ,z_{n})=z$$

Similar to Theorem 2, Theorem 7 can be proved.

**Theorem 8.** (Boundedness). Let *z*_*i*_ = (*A*_∅(*i*)_,*B*_*φ*(*i*)_) (*i* = 1,…,*n*) be a set of LZNs and *W* = (*w*_1_,*w*_2_,…,*w*_*n*_) is the weight vector of *z*_*i*_ (*i* = 1,…,*n*) such that *w*_*i*_∈[0,1] and $\sum_{i=1}^{n}w_{i}=1$, and let:

$$z^{-}=\min_{i}z_{i}=(A_{z^{-}},B_{z^{-}})=(\min_{i}\left(A_{\emptyset (i)}\right),\min_{i}\left(B_{\varphi (i)}\right))$$

$$z^{+}=\max_{i}z_{i}=(A_{z^{+}},B_{z^{+}})=(\max_{i}\left(A_{\emptyset (i)}\right),\max_{i}\left(B_{\varphi (i)}\right))$$

then,

$$z^{-}\leq {LZOWA}_{w}(z_{1},z_{2},\ldots ,z_{n})\leq z^{+}$$

Similar to Theorem 3, Theorem 8 can easily be proved.

**Theorem 9.** (Monotonicity). Let *z*_*i*_ = (*A*_∅(*i*)_,*B*_*φ*(*i*)_) (*i* = 1,…,*n*) and $z_{i}^{*}=(A_{\emptyset (i)}^{*},B_{\varphi (i)}^{*})\;(i=1,\ldots ,n)$ be two sets of LZNs and *W* = (*w*_1_,*w*_2_,…,*w*_*n*_) is the weight vector of *z*_*i*_ *and* $z_{i}^{*}$ (*i* = 1,…,*n*) such that *w*_*i*_∈[0,1] and $\sum_{i=1}^{n}w_{i}=1$. If $A_{\emptyset (i)}\leq A_{\emptyset (i)}^{*}$ and $B_{\varphi (i)}\leq B_{\varphi (i)}^{*}$, for all *i*, then,

$${LZOWA}_{w}(z_{1},z_{2},\ldots ,z_{n})\leq {LZOWA}_{w}(z_{1}^{*},z_{2}^{*},\ldots ,z_{n}^{*})$$

Similar to Theorem 4, Theorem 9 can easily be proved.

**Theorem 10.** Let *z*_*i*_ = (*A*_∅(*i*)_,*B*_*φ*(*i*)_) (*i* = 1,…,*n*) be a set of LZNs and *W* = (*w*_1_,*w*_2_,…,*w*_*n*_) is the weight vector of *z*_*i*_ (*i* = 1,…,*n*) such that *w*_*i*_∈[0,1] and $\sum_{i=1}^{n}w_{i}=1$. For each *q*>0, we can indicate:

$${LZOWA}_{w}(qz_{1},qz_{2},\ldots ,qz_{n})={qLZOWA}_{w}(z_{1},z_{2},\ldots ,z_{n})$$

Similar to Theorem 5, Theorem 10 can easily be proved.

It is noted that the LZOWA operator does not possess commutativity. For example, let $z_{1}=(s_{6},s_{3}^{\prime }),\;z_{2}=(s_{5},s_{4}^{\prime }),\;z_{3}=(s_{4},s_{3}^{\prime })$ and $z_{4}=(s_{3},s_{2}^{\prime })$, then ${LZOWA}_{w}(z_{1},z_{2},z_{3},z_{4})=(s_{4.5},s_{2.94}^{\prime })$ and ${LZOWA}_{w}(z_{4},z_{1},z_{3},z_{2})=(s_{4.31},s_{3.16}^{\prime })$ for *w* = (0.155,0.345,0.345,0.155).

In the following, the special case of LZOWA operator is presented:

If $W=(\frac{1}{n},\frac{1}{n},\ldots ,\frac{1}{n})$, then the LZOWA operator is reduced to linguistic Z-number ordered averaging (LZOA) operator, which is defined as follows:

$${LZOWA}_{w}(z_{1},z_{2},\ldots ,z_{n})=\frac{1}{n}\sum_{i=1}^{n}z_{\sigma (i)}$$

### 3.3. Linguistic Z-number hybrid weighted averaging (LZHWA) aggregation operator

The linguistic Z-number weighted averaging (LZWA) operator weights only the linguistic Z-number arguments, while the linguistic Z-number ordered weighted averaging (LZOWA) operator weights only the ordered situation of the linguistic Z-number values. In order to come over this limitation, a linguistic Z-number hybrid weighted averaging (LZHWA) operator is developed. The LZHWA operator weights both the linguistic Z-number arguments and their ordered situations.

**Definition 11.** Suppose *Z* = {*z*_*i*_ = (*A*_∅(*i*)_,*B*_*φ*(*i*)_)|*i* = 1,…,*n*} be a set of linguistic Z-numbers (LZNs), which have an associated vector *W* = (*w*_1_,*w*_2_,…,*w*_*n*_) such that *w*_*i*_∈[0,1] and $\sum_{i=1}^{n}w_{i}=1$. Then, linguistic Z-number hybrid weighted averaging (LZHWA) aggregation operator can be defined as follows:

$${LZHWA}_{\omega ,w}(z_{1},z_{2},\ldots ,z_{n})=\sum_{i=1}^{n}w_{i}{\dot{z}}_{\sigma (i)}$$ (20)

Where ${\dot{z}}_{\sigma (i)}$ is the *i*^*th*^ largest of the weighted Z-number ${\dot{z}}_{i}\;({\dot{z}}_{i}=n\omega_{i}z_{i}),$ *ω* = (*ω*_1_,*ω*_2_,…,*ω*_*n*_) is the weight vector of *z*_*i*_ (*i* = 1,…,*n*) such that *ω*_*i*_∈[0,1] and $\sum_{i=1}^{n}\omega_{i}=1$.

Based on the operations of linguistic Z-numbers represented in Definition 4, we can get the following result.

**Theorem 11.** Let *z*_*i*_ = (*A*_∅(*i*)_,*B*_*φ*(*i*)_) (*i* = 1,…,*n*) be a set of LZNs, which have an associated vector *W* = (*w*_1_,*w*_2_,…,*w*_*n*_) such that *w*_*i*_∈[0,1] and $\sum_{i=1}^{n}w_{i}=1$. Then, the aggregated result acquired based on LZHWA operator is also a LZN and is indicated as follows:

$${LZHWA}_{\omega ,w}(z_{1},z_{2},\ldots ,z_{n})=\left(f^{{*}^{-1}}\left(\sum_{i=1}^{n}w_{i}f^{*}({\dot{A}}_{\emptyset (\sigma (i))})\right),g^{{*}^{-1}}(\frac{\sum_{i=1}^{n}\left(w_{i}f^{*}({\dot{A}}_{\emptyset (\sigma (i))})\times g^{*}({\dot{B}}_{\varphi (\sigma (i))})\right)}{\sum_{i=1}^{n}w_{i}f^{*}({\dot{A}}_{\emptyset (\sigma (i))})})\right)$$ (21)

Where ${\dot{z}}_{\sigma (i)}$ is the *i*^*th*^ largest of the weighted Z-number ${\dot{z}}_{i}\;({\dot{z}}_{i}=n\omega_{i}z_{i}),$ *ω* = (*ω*_1_,*ω*_2_,…,*ω*_*n*_) is the weight vector of *z*_*i*_ (*i* = 1,…,*n*) such that *ω*_*i*_∈[0,1] and $\sum_{i=1}^{n}\omega_{i}=1$.

Similarly, Theorem 11 can easily be proven by using Definition 5 and the mathematical induction technique.

It is easy to prove that the LZHWA operator has the following properties.

**Theorem 12.** (Idempotency). Let *z*_*i*_ = (*A*_∅(*i*)_,*B*_*φ*(*i*)_) (*i* = 1,…,*n*) be a set of LZNs, which have an associated vector *W* = (*w*_1_,*w*_2_,…,*w*_*n*_) such that *w*_*i*_∈[0,1] and $\sum_{i=1}^{n}w_{i}=1$. If all *z*_*i*_ are equal, i.e., *z*_*i*_ = *z* = (*A*_∅(.)_,*B*_*φ*(.)_), for all *i*, then:

$${LZHWA}_{\omega ,w}(z_{1},z_{2},\ldots ,z_{n})=z$$

Where ${\dot{z}}_{\sigma (i)}$ is the *i*^*th*^ largest of the weighted Z-number ${\dot{z}}_{i}\;({\dot{z}}_{i}=n\omega_{i}z_{i}),$ *ω* = (*ω*_1_,*ω*_2_,…,*ω*_*n*_) is the weight vector of *z*_*i*_ (*i* = 1,…,*n*) such that *ω*_*i*_∈[0,1] and $\sum_{i=1}^{n}\omega_{i}=1$.

Similar to Theorem 2, Theorem 12 can easily be proved.

**Theorem 13.** (Boundedness). Let *z*_*i*_ = (*A*_∅(*i*)_,*B*_*φ*(*i*)_) (*i* = 1,…,*n*) be a set of LZNs and *W* = (*w*_1_,*w*_2_,…,*w*_*n*_) is the weight vector of *z*_*i*_ (*i* = 1,…,*n*) such that *w*_*i*_∈[0,1] and $\sum_{i=1}^{n}w_{i}=1$, and let:

$$z^{-}=\min_{i}z_{i}=(A_{z^{-}},B_{z^{-}})=(\min_{i}\left(A_{\emptyset (i)}\right),\min_{i}\left(B_{\varphi (i)}\right))$$

$$z^{+}=\max_{i}z_{i}=(A_{z^{+}},B_{z^{+}})=(\max_{i}\left(A_{\emptyset (i)}\right),\max_{i}\left(B_{\varphi (i)}\right))$$

then,

$$z^{-}\leq {LZHWA}_{w}(z_{1},z_{2},\ldots ,z_{n})\leq z^{+}$$

Similar to Theorem 3, Theorem 13 can easily be proved.

**Theorem 14.** (Monotonicity). Let *z*_*i*_ = (*A*_∅(*i*)_,*B*_*φ*(*i*)_) (*i* = 1,…,*n*) and $z_{i}^{*}=(A_{\emptyset (i)}^{*},B_{\varphi (i)}^{*})\;(i=1,\ldots ,n)$ be two sets of LZNs and *W* = (*w*_1_,*w*_2_,…,*w*_*n*_) is the weight vector of *z*_*i*_ *and* $z_{i}^{*}$ (*i* = 1,…,*n*) such that *w*_*i*_∈[0,1] and $\sum_{i=1}^{n}w_{i}=1$. If $A_{\emptyset (i)}\leq A_{\emptyset (i)}^{*}$ and $B_{\varphi (i)}\leq B_{\varphi (i)}^{*}$, for all *i*, then,

$${LZHWA}_{w}(z_{1},z_{2},\ldots ,z_{n})\leq {LZHWA}_{w}(z_{1}^{*},z_{2}^{*},\ldots ,z_{n}^{*})$$

Similar to Theorem 4, Theorem 14 can easily be proved.

**Theorem 15.** Let *z*_*i*_ = (*A*_∅(*i*)_,*B*_*φ*(*i*)_) (*i* = 1,…,*n*) be a set of LZNs, which have an associated vector *W* = (*w*_1_,*w*_2_,…,*w*_*n*_) such that *w*_*i*_∈[0,1] and $\sum_{i=1}^{n}w_{i}=1$. For each *q*>0, we can indicate:

$${LZHWA}_{\omega ,w}(qz_{1},qz_{2},\ldots ,qz_{n})={qLZHWA}_{\omega ,w}(z_{1},z_{2},\ldots ,z_{n})$$

Where ${\dot{z}}_{\sigma (i)}$ is the *i*^*th*^ largest of the weighted Z-number ${\dot{z}}_{i}\;({\dot{z}}_{i}=n\omega_{i}z_{i}),$ *ω* = (*ω*_1_,*ω*_2_,…,*ω*_*n*_) is the weight vector of *z*_*i*_ (*i* = 1,…,*n*) such that *ω*_*i*_∈[0,1] and $\sum_{i=1}^{n}\omega_{i}=1$.

Similar to Theorem 5, Theorem 15 can easily be proven.

In the following, the special case of LZHWA operator is presented:

If $W=(\frac{1}{n},\frac{1}{n},\ldots ,\frac{1}{n})$, then the LZHWA operator is reduced to linguistic Z-number averaging (LZOWA) operator.

It should be noted that the LZHWA operator does not possess commutativity. For example, let $z_{1}=(s_{6},s_{3}^{\prime }),\;z_{2}=(s_{5},s_{4}^{\prime }),\;z_{3}=(s_{4},s_{3}^{\prime })$ and $z_{4}=(s_{3},s_{2}^{\prime })$, then ${LZHWA}_{w}(z_{1},z_{2},z_{3},z_{4})=(s_{5.003},s_{3.23}^{\prime })$ and ${LZHWA}_{w}(z_{4},z_{1},z_{3},z_{2})=(s_{4.49},s_{2.92}^{\prime })$ for *w* = (0.155,0.345,0.345,0.155).

Case 1: If $W=(w_{1},w_{2},\ldots ,w_{n})=(\frac{1}{n},\frac{1}{n},\ldots ,\frac{1}{n})$, then the LZHWA operator is reduced to linguistic Z-number weighted averaging (LZWA) operator.

Proof. Suppose $W=(w_{1},w_{2},\ldots ,w_{n})=(\frac{1}{n},\frac{1}{n},\ldots ,\frac{1}{n})$, then

$${LZHWA}_{\omega ,w}(z_{1},z_{2},\ldots ,z_{n})=w_{1}{\dot{z}}_{\sigma (1)}+w_{2}{\dot{z}}_{\sigma (2)}+\ldots +w_{n}{\dot{z}}_{\sigma (n)}=\frac{1}{n}({\dot{z}}_{\sigma (1)}+{\dot{z}}_{\sigma (2)}+\ldots +{\dot{z}}_{\sigma (n)})=w_{1}z_{1}+w_{2}z_{2}+\ldots +w_{n}z_{n}={LZWA}_{w}(z_{1},z_{2},\ldots ,z_{n})∎$$

Case 2: If $\omega =(\omega_{1},\omega_{2},\ldots ,\omega_{n})=\left(\frac{1}{n},\frac{1}{n},\ldots ,\frac{1}{n}\right)$, then the LZHWA operator is reduced to linguistic Z-number ordered weighted averaging (LZOWA) operator.

Proof. Suppose $\omega =(\omega_{1},\omega_{2},\ldots ,\omega_{n})=\left(\frac{1}{n},\frac{1}{n},\ldots ,\frac{1}{n}\right)$, then ${\dot{z}}_{\sigma (i)}=z_{i},\;i=1,2,\ldots ,n$, thus

$${LZHWA}_{\omega ,w}(z_{1},z_{2},\ldots ,z_{n})=w_{1}{\dot{z}}_{\sigma (1)}+w_{2}{\dot{z}}_{\sigma (2)}+\cdots +w_{n}{\dot{z}}_{\sigma (n)}=w_{1}z_{1}+w_{2}z_{2}+\cdots +w_{n}z_{n}={LZOWA}_{w}(z_{1},z_{2},\ldots ,z_{n})∎$$

Based on the special cases of LZHWA operator, it can be seen that the LZHWA operator can generalize both the LZWA operator and LZOWA operator.

## 4. Portfolio selection based on aggregation operators under linguistic Z-number environment

**Fig 1:**
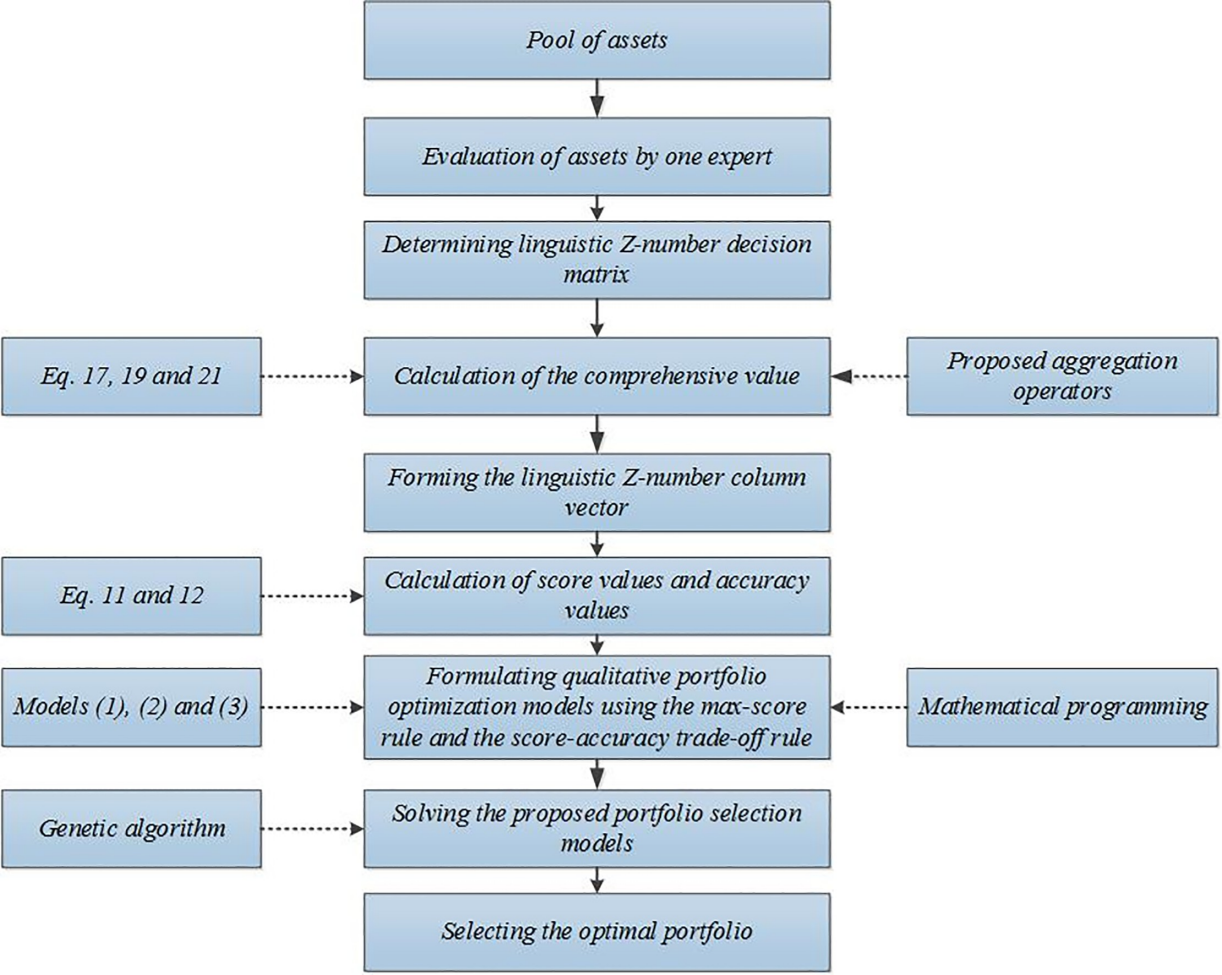
Flowchart for the qualitative portfolio selection based on the linguistic Z-number aggregation operators.

In this section, a multi-stage qualitative approach is presented to model the qualitative portfolio problems based on the proposed aggregation operators under the linguistic Z-number environment. One expert evaluates the performance of assets according to his/her knowledge and judgment, which can be used to represent the uncertainty and the reliability of data, simultaneously. The proposed aggregation operators are powerful tools to incorporate expert’s knowledge under the linguistic Z-number environment. Fig 1 shows a total schematic of the proposed approach.

The qualitative approach has three steps. In the first step, we evaluate the performance of each asset according to the financial criteria and an expert’s opinion. In the second step, the proposed aggregation operators calculate the comprehensive values of each asset. In the third step, we propose three qualitative portfolio models based on the preferences of the experts and the investors.

### 4.1. The first step: Assessment of assets

Suppose that there are *n* risky assets as {*x*_1_,*x*_2_,…,*x*_*n*_} that investors can allocate their wealth to them. These assets are evaluated by expert based on *m* financial criteria {*f*_1_,*f*_2_,…,*f*_*m*_}. The assessment data can be described by the linguistic Z-numbers as *z*_*ij*_ (*i* = 1,…,*n and j* = 1,…,*m*), and can be represented as a linguistic Z-number matrix as *Z* = [*z*_*ij*_]_*n*×*m*_.

### 4.2. The second step: Calculating the comprehensive value of each asset and its corresponding score

In this step, the comprehensive values of each asset are computed based on the proposed aggregation operators as ${\bar{z}}_{i}=({\bar{A}}_{\emptyset (i)},{\bar{B}}_{\varphi (i)})$. The aggregated values are calculated under the proposed aggregation operators as follows:

$${\bar{z}}_{i}={LZWA}_{w}(z_{i1},\ldots ,z_{im})$$

Or

$${\bar{z}}_{i}={LZOWA}_{w}(z_{i1},\ldots ,z_{im})$$

Or

$${\bar{z}}_{i}={LZHWA}_{\omega ,w}(z_{i1},\ldots ,z_{im})$$

Consequently, the linguistic Z-number matrix *Z* = [*z*_*ij*_]_*n*×*m*_ can be converted into a qualitative column vector $\bar{Z}={\left[{\bar{z}}_{i}\right]}_{n\times 1}$ by aggregating all the linguistic Z-numbers on one line. Then, the score values and the accuracy values are obtained according to Definition 5 as follows:

$$S({\bar{z}}_{i})=f^{*}({\bar{A}}_{\emptyset (i)})\times g^{*}({\bar{B}}_{\varphi (i)})$$

$$A({\bar{z}}_{i})=f^{*}({\bar{A}}_{\emptyset (i)})\times \left(1-g^{*}({\bar{B}}_{\varphi (i)})\right)$$

The resulted values can be represented as two column vectors $S={\left[S({\bar{z}}_{i})\right]}_{n\times 1},\;A={\left[A({\bar{z}}_{i})\right]}_{n\times 1}$.

### 4.3. The third step: Portfolio selection based on aggregation operators under linguistic Z-number environment

In the third step, three portfolio models are presented to assign the best assets based on the proposed aggregation operators under the linguistic Z-number environment. These models can be considered as the substitute methods of creating more diversified portfolios. In the proposed qualitative portfolio models, the diversification of portfolio can be handled by considering entropy function or by using lower and upper bounds of a fraction of the investment budget in each asset along with a predefined number of assets that can be allocated to the selected portfolio. Hong and Choi [59] showed that the relation between the score function and the accuracy function is equivalent to the relation between the mean and variance of quantitative data in statistics. Therefore, the score and accuracy values can be employed to measure the expected return and risk values of the portfolios under linguistic Z-number environment. In the following, three linguistic Z-number portfolio models are formulated according to the max-score rule and the score-accuracy trade-off rule such that the portfolios can be chosen by maximizing score function as an objective function along with an admissible level of risk as a constraint. Moreover, these qualitative optimization models can examine the trade-off among the score and accuracy values. Firstly, the objective function and constraints in all the three proposed qualitative portfolio models are introduced as follows:

- **Objective function**

According to the max-score rule, the objective function can be defined as follows:

$$Max\;\sum_{i=1}^{n}((S({\bar{z}}_{i}))x_{i})=\sum_{i=1}^{n}\left((f^{*}({\bar{A}}_{\emptyset (i)})\times g^{*}({\bar{B}}_{\varphi (i)}))x_{i}\right)$$ (22)

- Cardinality constraint

$$\sum_{i=1}^{s}x_{i}=1$$ (23)

$$\sum_{i=1}^{s}y_{i}=h$$ (24)

$$l_{i}y_{i}\leq x_{i}\leq u_{i}y_{i},\;i=1,\ldots ,n$$ (25)

$$y_{i}\in \{0,1\},\;i=1,\ldots ,n$$ (26)

$$x_{i}\geq 0,\;i=1,\ldots ,n$$ (27)

Constraint (23) is the budget constraint. Constraint (24), which is called cardinality constraint, guarantees that the portfolio is limited to maintain a predefined number of securities such as *h*. *l*_*i*_ (≥0) is the minimum portion of total capital which can be allocated to the *i*^*th*^ asset and *u*_*i*_ (0≤*l*_*i*_≤*u*_*i*_) is the maximum portion of total investment which can be devoted to the *i*^*th*^ asset. Let *x*_*i*_ is the weight of the *i*^*th*^ asset in the portfolio and *y*_*i*_ is a binary variable which is equal to one when the corresponding asset is allocated to portfolio, otherwise, it is zero. Eventually, the prohibition of short selling is shown constraint (27).

- **Entropy constraint**

To achieve more diversified portfolio and capture the topic of diversification, the following concave entropy function, which is presented by Shannon [60], can be applied:

$$E(x)=-\sum_{i=1}^{n}x_{i}\ln x_{i}$$ (28)

It should be mentioned that the entropy function (*E*(*x*)) takes its maximum values (ln *n*) when $x_{i}=\frac{1}{n}$. Moreover, if *x*_*i*_ = 1 for one asset and *x*_*i*_ = 0 for other assets, then *E*(*x*) = 0. Hence, a suitable measure of disorder in a probability distribution is provided by this entropy function. Consequently, it can be found that this entropy function is proper for handling the diversification of portfolio. To obtain a suitable level of diversification, the following constraint is applied:

$$-\sum_{i=1}^{n}x_{i}\ln x_{i}\geq \delta \;0\leq \delta \leq \ln n$$ (29)

- **Risk constraint**

As discussed, the accuracy function of linguistic Z-numbers can be applied to control the risk of the portfolio. When the investor selects the desired level of risk, a risk constraint can be imposed on the asset allocation problem. The risk constraint using the accuracy function is defined as follows:

$$\sum_{i=1}^{n}\left((A({\bar{Z}}_{i}))x_{i}\right)=\sum_{i=1}^{n}\left((f^{*}({\bar{A}}_{\emptyset (i)})\times (1-g^{*}({\bar{B}}_{\varphi (i)})))x_{i}\right)\geq \vartheta$$ (30)

where $\vartheta \in [0,{max}_{1\leq i\leq n}(A({\bar{Z}}_{i}))]$ is a minimum admissible level of the risk value. The *ϑ*-value is determined by investors or fund managers. Three cases may occur for determining the *ϑ*-value:

1. If $\vartheta >{max}_{1\leq i\leq n}(A({\bar{Z}}_{i}))$, then, no portfolio is constructed because no feasible solution can be detected.
2. If $\vartheta ={max}_{1\leq i\leq n}(A({\bar{Z}}_{i}))$, then, only one portfolio can be generated.
3. If $0\leq \vartheta \leq {max}_{1\leq i\leq n}(A({\bar{Z}}_{i}))$, then, the greater the *ϑ*-value, the greater the effect of admissible level of the risk value in the portfolio performance.

Now, we propose three hybrid qualitative portfolio models. The first model is based on the max-score rule along with the cardinality constraint. The second model is based on the max-score rule along with the entropy constraint. The investors who are intending to achieve the maximum return can use these two proposed qualitative portfolio models. Hence, these models are suitable for general investors. The third model is developed according to the score-accuracy trade-off rule with consideration of the entropy constraint. The investors who want to achieve the maximum return along with a desired risk level can use the third model. Therefore, the third model is appropriate for the risky investors.

#### 4.3.1. Model (1): The qualitative portfolio optimization model based on the max-score rule along with the cardinality constraint

The max-score rule is simple and proper for describing the investment goals of general investors. Hence, the corresponding qualitative portfolio model (Model (1)) is developed as follows:

$$\mathrm{Model}\;(1):\;Max\;\sum_{i=1}^{n}((S({\bar{z}}_{i}))x_{i})=\sum_{i=1}^{n}(\left(f^{*}({\bar{A}}_{\emptyset (i)})\times g^{*}({\bar{B}}_{\varphi (i)}))x_{i}\right)$$

S.t.

$$\sum_{i=1}^{s}x_{i}=1$$

$$\sum_{i=1}^{s}y_{i}=h$$

$$l_{i}y_{i}\leq x_{i}\leq u_{i}y_{i},\;i=1,\ldots ,n$$

$$y_{i}\in \{0,1\},\;i=1,\ldots ,n$$

$$x_{i}\geq 0,\;i=1,\ldots ,n$$

Model (1) is a mixed integer linear convex optimization problem. The linearity structure of Model (1) maintains a very significant feature that every local optimal point is also a global optimal point. This feature guarantees that the obtained solution from Model (1) is optimal.

#### 4.3.2. Model (2): The qualitative portfolio optimization model based on the max-score rule along with the entropy constraint

Similar to Model (1), the second qualitative portfolio model (Model (2)) is developed by considering the entropy constraint as follows:

$$\mathrm{Model}\;(2):\;Max\;\sum_{i=1}^{n}((S({\bar{z}}_{i}))x_{i})=\sum_{i=1}^{n}\left((f^{*}({\bar{A}}_{\emptyset (i)})\times g^{*}({\bar{B}}_{\varphi (i)}))x_{i}\right)$$

S.t.

$$\sum_{i=1}^{s}x_{i}=1$$

$$-\sum_{i=1}^{n}x_{i}\;\ln\;x_{i}\geq \delta \;0\leq \delta \leq \;\ln\;n\;and\;i=1,\ldots ,n$$

$$x_{i}\geq 0,\;i=1,\ldots ,n$$

Model (2) is a nonlinear convex optimization problem. Solving the nonlinear optimization models can be computationally hard for medium or large-size problems.

**Proposition 1.** Model (2) is a convex optimization problem, i.e. it includes the maximization of a linear function subject to convex constraints.

Proof. The objective function $\sum_{i=1}^{n}((S({\bar{z}}_{i}))x_{i})=\sum_{i=1}^{n}\left((f^{*}({\bar{A}}_{\emptyset (i)})\times g^{*}({\bar{B}}_{\varphi (i)}))x_{i}\right)$ of Model (2) is a linear function which is both convex and concave. The constraint $\sum_{i=1}^{s}x_{i}=1$ is a linear function and thus, it is convex. It may be mentioned that the entropy function $E(x)=-\sum_{i=1}^{n}x_{i}\;\ln\;x_{i}$ is a concave function. Therefore, $-E(x)=\sum_{i=1}^{n}x_{i}\;\ln\;x_{i}$ is a convex function and hence, the constraint $-\sum_{i=1}^{n}x_{i}\;\ln\;x_{i}\geq \delta$ which can be revised as $\sum_{i=1}^{n}x_{i}\;\ln\;x_{i}+\delta \geq 0,\;0\leq \delta \leq \ln\;n\;and\;i=1,\ldots ,n$ is also convex. The constraint *x*_*i*_≥0 is also a linear function and it can be rewritten as −*x*_*i*_≤0,*i* = 1,2,…,*n*, thus, it is convex. Consequently, Model (2) is a convex optimization problem.

#### 4.3.3. Model (3): The qualitative portfolio optimization model based on the score-accuracy trade-off rule along with the entropy constraint

The score-accuracy trade-off rule is appropriate for the risky investors who are intending to achieve the maximum return along with the limited level of risk. Hence, the corresponding qualitative portfolio model (Model (3)) is developed as follows:

$$\mathrm{Model}\;(3):\;Max\;\sum_{i=1}^{n}((S({\bar{z}}_{i}))x_{i})=\sum_{i=1}^{n}\left((f^{*}({\bar{A}}_{\emptyset (i)})\times g^{*}({\bar{B}}_{\varphi (i)}))x_{i}\right)$$

S.t.

$$\sum_{i=1}^{n}\left((A({\bar{Z}}_{i}))x_{i}\right)=\sum_{i=1}^{n}\left((f^{*}({\bar{A}}_{\emptyset (i)})\times (1-g^{*}({\bar{B}}_{\varphi (i)})))x_{i}\right)\geq \vartheta$$

$$\sum_{i=1}^{s}x_{i}=1$$

$$-\sum_{i=1}^{n}x_{i}\;\ln x_{i}\geq \delta \;0\leq \delta \leq \ln\;n\;and\;i=1,\ldots ,n$$

$$x_{i}\geq 0,\;i=1,\ldots ,n$$

Model (3) is also a nonlinear convex optimization problem. Therefore, solving this model can be computationally difficult for medium or large-size problems.

**Proposition 2.** Model (3) is a convex optimization problem, i.e. it includes the maximization of a linear function subject to convex constraints.

Proof. The objective function $\sum_{i=1}^{n}((S({\bar{z}}_{i}))x_{i})=\sum_{i=1}^{n}\left((f^{*}({\bar{A}}_{\emptyset (i)})\times g^{*}({\bar{B}}_{\varphi (i)}))x_{i}\right)$ of Model (3) is a linear function which is both convex and concave. The constraint $\sum_{i=1}^{n}\left((A({\bar{Z}}_{i}))x_{i}\right)=\sum_{i=1}^{n}\left((f^{*}({\bar{A}}_{\emptyset (i)})\times (1-g^{*}({\bar{B}}_{\varphi (i)})))x_{i}\right)\geq \vartheta$ is a linear function and can be rewritten as $-\sum_{i=1}^{n}\left((f^{*}({\bar{A}}_{\emptyset (i)})\times (1-g^{*}({\bar{B}}_{\varphi (i)})))x_{i}\right)+\vartheta \leq 0$, thus, it can be taken as convex function. The constraint $\sum_{i=1}^{s}x_{i}=1$ is a linear function and thus, it is convex. It may be mentioned that the entropy function $E(x)=-\sum_{i=1}^{n}x_{i}\;\ln\;x_{i}$ is a concave function. Therefore, $-E(x)=\sum_{i=1}^{n}x_{i}\;\ln\;x_{i}$ is a convex function and hence, the constraint $-\sum_{i=1}^{n}x_{i}\;\ln\;x_{i}\geq \delta$ which can be revised as $\sum_{i=1}^{n}x_{i}\;\ln\;x_{i}+\delta \geq 0,\;0\leq \delta \leq \ln\;n\;and\;i=1,\ldots ,n$ is also convex. The constraint *x*_*i*_≥0 is also a linear function and it can be rewritten as −*x*_*i*_≤0,*i* = 1,2,…,*n*, thus, it is convex. Consequently, Model (3) is a convex optimization problem.

## 5. Case study and computational results

In this section, The Tehran stock exchange market (TSE) in Iran has been used as the information source. The necessary data is available through the site of Tehran stock exchange market [61]. TSE is Iran's largest stock exchange, which first opened in 1967. In May 2012, TSE listed 339 companies with a combined market capitalization of US$104.21 billion. There are 37 industries such as the automotive, telecommunications, petrochemical, mining, steel iron, copper, banking, and financial mediation at the stock market in TSE. At the end of each season, the department of information of the Tehran stock exchange market reveals the name of 50 best corporations. These corporations are selected by certain criteria. We choose 20 firms with the best performance in the latest financial statement from 20 January 2019 to 20 June 2019 to validate the proposed qualitative approach.

### 5.1. The first step: Assessment of assets

We consider the information of these 20 firms as input of a fuzzy system to assess financial criteria. In this step, to describe the information, linguistic variables in Tables 1 and 2 are used for evaluating the performance of each asset concerning some given criteria. On the other hand, the expert’s experience can be indicated by using fuzzy if-then rules [62]. Thus, this study uses these rules to better evaluate the performance of firms concerning the given criteria.

**Table 1:** Linguistic variables for evaluating financial performance of firms (S).

Very very low (VVL)	Almost high (AH)
Very low (VL)	High (H)
Low (L)	Very high (VH)
Almost medium (AM)	Perfect (P)
Medium (M)	

**Table 2:** Linguistic variables for evaluating expert’s reliability (S′).

Seldom (S)	Regularly (R)
Occasionally (O)	Usually (U)
Frequently (F)	

In this study, suppose that investors are intending to compare the performance of these 20 firms using four financial criteria: short term returns (STR), long term returns (LTR), external reputation (ER) and liquidity (L). Then, one expert evaluates the performance of each firm concerning these mentioned criteria, and he/she expresses his/her opinions using the linguistic terms provided in Tables 1 and 2. The linguistic terms set *S* = {*s*_0_,*s*_1_,*s*_2_,*s*_3_,*s*_4_,*s*_5_,*s*_6_,*s*_7_,*s*_8_}, which is shown in Table 1, can be applied to assess the performance of firms with respect to the mentioned criteria. Moreover, the linguistic terms set $S^{\prime }=\{s_{0}^{\prime },s_{1}^{\prime },s_{2}^{\prime },s_{3}^{\prime },s_{4}^{\prime }\}$, which is indicated in Table 2, can be used to represent the reliability measure of the related information.

Thus, the performance of the firms {*x*_1_,*x*_2_,…,*x*_20_} is evaluated with respect to the given criteria by one expert. The evaluation information is described by the linguistic Z-numbers as *z*_*ij*_ (*i* = 1,…,20,*j* = 1,2,3,4) and linguistic Z-number matrix *Z* = [*z*_*ij*_]_20×4_ is generated based on the *z*_*ij*_. This matrix represents the performance of assets with respect to the given criteria. The results of the evaluation are displayed in Tables 3. Expert’s opinions are simulated based on the evaluation information. Therefore, the performance of the proposed qualitative portfolio models can efficiently be examined.

**Table 3:** The evaluation information of firms which is represented by LZNs.

Asset ID	Short-term return	Long-term return	External reputation	Liquidity	Asset ID	Short-term return	Long-term return	External reputation	Liquidity
1	$\left(s_{6},s_{3}^{\prime }\right)$	$\left(s_{5},s_{4}^{\prime }\right)$	$\left(s_{4},s_{3}^{\prime }\right)$	$\left(s_{5},s_{4}^{\prime }\right)$	11	$\left(s_{5},s_{1}^{\prime }\right)$	$\left(s_{3},s_{4}^{\prime }\right)$	$\left(s_{5},s_{4}^{\prime }\right)$	$\left(s_{6},s_{1}^{\prime }\right)$
2	$\left(s_{7},s_{3}^{\prime }\right)$	$\left(s_{4},s_{4}^{\prime }\right)$	$\left(s_{2},s_{3}^{\prime }\right)$	$\left(s_{6},s_{3}^{\prime }\right)$	12	$\left(s_{1},s_{1}^{\prime }\right)$	$\left(s_{3},s_{2}^{\prime }\right)$	$\left(s_{5},s_{3}^{\prime }\right)$	$\left(s_{5},s_{1}^{\prime }\right)$
3	$\left(s_{5},s_{1}^{\prime }\right)$	$\left(s_{3},s_{2}^{\prime }\right)$	$\left(s_{6},s_{3}^{\prime }\right)$	$\left(s_{5},s_{2}^{\prime }\right)$	13	$\left(s_{6},s_{2}^{\prime }\right)$	$\left(s_{5},s_{3}^{\prime }\right)$	$\left(s_{2},s_{4}^{\prime }\right)$	$\left(s_{5},s_{3}^{\prime }\right)$
4	$\left(s_{2},s_{4}^{\prime }\right)$	$\left(s_{4},s_{3}^{\prime }\right)$	$\left(s_{6},s_{4}^{\prime }\right)$	$\left(s_{3},s_{3}^{\prime }\right)$	14	$\left(s_{6},s_{4}^{\prime }\right)$	$\left(s_{4},s_{3}^{\prime }\right)$	$\left(s_{4},s_{4}^{\prime }\right)$	$\left(s_{5},s_{3}^{\prime }\right)$
5	$\left(s_{3},s_{2}^{\prime }\right)$	$\left(s_{1},s_{2}^{\prime }\right)$	$\left(s_{5},s_{3}^{\prime }\right)$	$\left(s_{5},s_{1}^{\prime }\right)$	15	$\left(s_{5},s_{1}^{\prime }\right)$	$\left(s_{2},s_{4}^{\prime }\right)$	$\left(s_{6},s_{4}^{\prime }\right)$	$\left(s_{2},s_{3}^{\prime }\right)$
6	$\left(s_{4},s_{4}^{\prime }\right)$	$\left(s_{5},s_{2}^{\prime }\right)$	$\left(s_{4},s_{3}^{\prime }\right)$	$\left(s_{4},s_{2}^{\prime }\right)$	16	$\left(s_{7},s_{4}^{\prime }\right)$	$\left(s_{3},s_{4}^{\prime }\right)$	$\left(s_{4},s_{3}^{\prime }\right)$	$\left(s_{5},s_{3}^{\prime }\right)$
7	$\left(s_{7},s_{3}^{\prime }\right)$	$\left(s_{5},s_{4}^{\prime }\right)$	$\left(s_{3},s_{4}^{\prime }\right)$	$\left(s_{6},s_{3}^{\prime }\right)$	17	$\left(s_{4},s_{4}^{\prime }\right)$	$\left(s_{6},s_{3}^{\prime }\right)$	$\left(s_{5},s_{2}^{\prime }\right)$	$\left(s_{6},s_{4}^{\prime }\right)$
8	$\left(s_{3},s_{4}^{\prime }\right)$	$\left(s_{4},s_{3}^{\prime }\right)$	$\left(s_{5},s_{3}^{\prime }\right)$	$\left(s_{3},s_{4}^{\prime }\right)$	18	$\left(s_{5},s_{1}^{\prime }\right)$	$\left(s_{3},s_{2}^{\prime }\right)$	$\left(s_{5},s_{4}^{\prime }\right)$	$\left(s_{1},s_{4}^{\prime }\right)$
9	$\left(s_{7},s_{4}^{\prime }\right)$	$\left(s_{6},s_{3}^{\prime }\right)$	$\left(s_{4},s_{4}^{\prime }\right)$	$\left(s_{7},s_{4}^{\prime }\right)$	19	$\left(s_{3},s_{4}^{\prime }\right)$	$\left(s_{7},s_{2}^{\prime }\right)$	$\left(s_{2},s_{2}^{\prime }\right)$	$\left(s_{6},s_{3}^{\prime }\right)$
10	$\left(s_{3},s_{4}^{\prime }\right)$	$\left(s_{2},s_{2}^{\prime }\right)$	$\left(s_{6},s_{3}^{\prime }\right)$	$\left(s_{4},s_{1}^{\prime }\right)$	20	$\left(s_{1},s_{4}^{\prime }\right)$	$\left(s_{3},s_{3}^{\prime }\right)$	$\left(s_{7},s_{3}^{\prime }\right)$	$\left(s_{2},s_{2}^{\prime }\right)$

### 5.2. The second step: Computing the comprehensive value of each alternative and its corresponding score and accuracy values

In this step, linguistic Z-number matrix *Z* = [*z*_*ij*_]_20×4_ can be converted into the qualitative column vector $\bar{Z}={\left[{\bar{z}}_{i}\right]}_{20\times 1}$ by fusing all the linguistic Z-numbers on one line based on LZWA operator or LZOWA operator or LZHWA operator. Suppose that *W* = (0.155,0.345,0.345,0.155), which is resulted by the normal distribution based on Xu’s method [63], is the weighting vector of the proposed operators, and *ω* = (0.28,0.24,0.22,0.26) is the weighting vector of *z*_*ij*_ (*i* = 1,…,20 *and j* = 1,2,3,4) in LZHWA operator. Also, suppose that f*(*x*_*i*_) = *LSF* 1 and g*(*x*_*i*_) = *LSF* 1. Therefore, the qualitative vectors $\bar{Z}={\left[{\bar{z}}_{i}\right]}_{20\times 1}$, which are shown in Tables 4–6, are calculated based on LZWA operator, LZOWA operator and LZHWA operator, respectively. Then, the score values and the accuracy values are obtained according to Definition 5, and the resulted values are reported in Tables 4–6. To achieve this aim, the linguistic Z-number information resulted shown in Table 3 is aggregated using Eqs 17, 19 and 21. Typically for asset 1, the corresponding comprehensive value based on the proposed operators is calculated using Eqs 17, 19 and 21 as follows.

- $LZWA(\left(s_{6},s_{3}^{\prime }),(s_{5},s_{4}^{\prime }),(s_{4},s_{3}^{\prime }),(s_{5},s_{4}^{\prime }\right))=(s_{4.81},s_{3.52}^{\prime })=Z_{1}^{\prime }$

and the corresponding score and accuracy values are computed using Eqs 11 and 12 as follows:

$$S(Z_{1}^{\prime })=0.601\times 0.879=0.529\;\mathrm{and}\;A(Z_{1}^{\prime })=0.601\times 0.12=0.0721$$

- $LZOWA(\left(s_{6},s_{3}^{\prime }),(s_{5},s_{4}^{\prime }),(s_{4},s_{3}^{\prime }),(s_{5},s_{4}^{\prime }\right))=(s_{5.19},s_{3.482}^{\prime })=Z_{2}^{\prime }$

and the corresponding score and accuracy values are computed using Eqs 11 and 12 as follows:

$$S(Z_{2}^{\prime })=0.648\times 0.88=0.565\;\mathrm{and}\;A(Z_{2}^{\prime })=0.648\times 0.129=0.084$$

- $LZHWA(\left(s_{6},s_{3}^{\prime }),(s_{5},s_{4}^{\prime }),(s_{4},s_{3}^{\prime }),(s_{5},s_{4}^{\prime }\right))=(s_{5.326},s_{3.462}^{\prime })=Z_{3}^{\prime }$

and the corresponding score and accuracy values are computed using Eqs 11 and 12 as follow:

$$S(Z_{3}^{\prime })=0.665\times 0.865=0.576\;\mathrm{and}\;A(Z_{3}^{\prime })=0.665\times 0.134=0.089$$

Similarly, the aggregated results and their corresponding score and accuracy values for all assets are calculated under the proposed operators and shown in Tables 4–6.

**Table 4:** The aggregated values and their corresponding score and accuracy values which resulted by LZWA operator.

Asset ID	Aggregated value	Score value	Accuracy value
1	$\left(s_{4.81},s_{3.52}^{\prime }\right)$	0.529	0.072
2	$\left(s_{4.43},s_{3.08}^{\prime }\right)$	0.426	0.128
3	$\left(s_{4.655},s_{2.28}^{\prime }\right)$	0.331	0.250
4	$\left(s_{4.225},s_{3.56}^{\prime }\right)$	0.470	0.058
5	$\left(s_{3.31},s_{2.29}^{\prime }\right)$	0.237	0.177
6	$\left(s_{4.345},s_{2.6}^{\prime }\right)$	0.353	0.190
7	$\left(s_{4.775},s_{3.58}^{\prime }\right)$	0.534	0.063
8	$\left(s_{4.035},s_{3.23}^{\prime }\right)$	0.407	0.097
9	$\left(s_{5.62},s_{3.63}^{\prime }\right)$	0.638	0.065
10	$\left(s_{3.845},s_{2.62}^{\prime }\right)$	0.315	0.166
11	$\left(s_{4.465},s_{2.85}^{\prime }\right)$	0.398	0.160
12	$\left(s_{3.69},s_{2.22}^{\prime }\right)$	0.255	0.206
13	$\left(s_{4.12},s_{2.94}^{\prime }\right)$	0.379	0.136
14	$\left(s_{4.465},s_{3.52}^{\prime }\right)$	0.491	0.067
15	$\left(s_{3.845},s_{3.31}^{\prime }\right)$	0.398	0.082
16	$\left(s_{4.275},s_{3.5}^{\prime }\right)$	0.467	0.067
17	$\left(s_{5,345},s_{2.97}^{\prime }\right)$	0.496	0.173
18	$\left(s_{3.69},s_{2.81}^{\prime }\right)$	0.324	0.137
19	$\left(s_{4.5},s_{2.41}^{\prime }\right)$	0.339	0.223
20	$\left(s_{3.915},s_{2.96}^{\prime }\right)$	0.362	0.127

**Table 5:** The aggregated values and their corresponding score and accuracy values which resulted by LZOWA operator.

Asset ID	Aggregated value	Score value	Accuracy value
1	$\left(s_{5.19},s_{3.482}^{\prime }\right)$	0.565	0.084
2	$\left(s_{5},s_{3.183}^{\prime }\right)$	0.497	0.128
3	$\left(s_{4.465},s_{2.035}^{\prime }\right)$	0.284	0.274
4	$\left(s_{3.655},s_{3.339}^{\prime }\right)$	0.381	0.075
5	$\left(s_{3.69},s_{1.743}^{\prime }\right)$	0.201	0.260
6	$\left(s_{4.345},s_{2.603}^{\prime }\right)$	0.353	0.190
7	$\left(s_{5.345},s_{3.41}^{\prime }\right)$	0.570	0.099
8	$\left(s_{3.655},s_{3.41}^{\prime }\right)$	0.390	0.067
9	$\left(s_{6.19},s_{3.66}^{\prime }\right)$	0.709	0.065
10	$\left(s_{3.655},s_{2.443}^{\prime }\right)$	0.279	0.178
11	$\left(s_{4.655},s_{2.166}^{\prime }\right)$	0.315	0.267
12	$\left(s_{3.69},s_{1.701}^{\prime }\right)$	0.196	0.265
13	$\left(s_{4.88},s_{2.639}^{\prime }\right)$	0.403	0.208
14	$\left(s_{4.655},s_{3.496}^{\prime }\right)$	0.509	0.073
15	$\left(s_{3.085},s_{3.023}^{\prime }\right)$	0.291	0.094
16	$\left(s_{4.655},s_{3.333}^{\prime }\right)$	0.485	0.097
17	$\left(s_{5.155},s_{3.298}^{\prime }\right)$	0.531	0.113
18	$\left(s_{3.69},s_{2.073}^{\prime }\right)$	0.235	0.226
19	$\left(s_{4.69},s_{2.64}^{\prime }\right)$	0.387	0.199
20	$\left(s_{2.965},s_{2.82}^{\prime }\right)$	0.261	0.109

**Table 6:** The aggregated values and their corresponding score and accuracy values which resulted by LZHWA operator.

Asset ID	Aggregated value	Score value	Accuracy value
1	$\left(s_{5.326},s_{3.462}^{\prime }\right)$	0.576	0.089
2	$\left(s_{5.102},s_{3.179}^{\prime }\right)$	0.507	0.131
3	$\left(s_{4.474},s_{1.989}^{\prime }\right)$	0.278	0.281
4	$\left(s_{3.566},s_{3.327}^{\prime }\right)$	0.371	0.075
5	$\left(s_{3.748},s_{1.706}^{\prime }\right)$	0.202	0.271
6	$\left(s_{4.209},s_{2.618}^{\prime }\right)$	0.344	0.182
7	$\left(s_{5.433},s_{3.38}^{\prime }\right)$	0.574	0.105
8	$\left(s_{3.71},s_{3.43}^{\prime }\right)$	0.398	0.066
9	$\left(s_{6.259},s_{3.683}^{\prime }\right)$	0.720	0.062
10	$\left(s_{3.71},s_{2.459}^{\prime }\right)$	0.285	0.179
11	$\left(s_{4.696},s_{2.07}^{\prime }\right)$	0.304	0.283
12	$\left(s_{3.643},s_{1.647}^{\prime }\right)$	0.188	0.268
13	$\left(s_{5.053},s_{2.595}^{\prime }\right)$	0.410	0.222
14	$\left(s_{4.645},s_{3.486}^{\prime }\right)$	0.506	0.075
15	$\left(s_{3.066},s_{2.917}^{\prime }\right)$	0.280	0.104
16	$\left(s_{4.548},s_{3.486}^{\prime }\right)$	0.495	0.073
17	$\left(s_{5.182},s_{3.353}^{\prime }\right)$	0.543	0.105
18	$\left(s_{3.768},s_{1.935}^{\prime }\right)$	0.228	0.243
19	$\left(s_{4.717},s_{2.696}^{\prime }\right)$	0.398	0.192
20	$\left(s_{2.657},s_{3.024}^{\prime }\right)$	0.251	0.081

By using the score and accuracy values reported in Tables 4–6, the ranking of assets according to Definition (5) in the descending order is as follows:

- LZWA operator

$$x_{9}{>x}_{7}>x_{1}>x_{17}>x_{14}>x_{4}>x_{16}>x_{2}>x_{8}>x_{11}>x_{15}>x_{13}>x_{20}>x_{6}>x_{19}>x_{3}>x_{18}>x_{10}>x_{12}>x_{5}$$

- LZOWA operator

$$x_{9}{>x}_{7}>x_{1}>x_{17}>x_{14}>x_{2}>x_{16}>x_{13}>x_{19}>x_{8}>x_{4}>x_{6}>x_{11}>x_{15}>x_{3}>x_{10}>x_{20}>x_{18}>x_{5}>x_{12}$$

- LZHWA operator

$$x_{9}{>x}_{7}>x_{1}>x_{17}>x_{2}>x_{14}>x_{16}>x_{13}>x_{19}>x_{8}>x_{4}>x_{6}>x_{11}>x_{10}>x_{15}>x_{3}>x_{20}>x_{18}>x_{5}>x_{12}$$

As obvious, the impact of reliability measures is reflected in the ranking results under all proposed operators. For example, the rank of the first asset (*x*_1_) is higher than the rank of 13^*th*^ asset (*x*_13_) under all operators because of the high value of reliability.

### 5.3. The third step: Portfolio selection based on aggregation operators under linguistic Z-number environment

In this step, Model (1), Model (2) and Model (3) are applied to formulate the qualitative portfolio problem. These models can choose the optimal combinations of assets according to the preferences of investors under linguistic Z-number environment. The proposed models are mixed-integer programming models. Speranza [64] indicated that the computational complexity of the mixed-integer linear programming models (MILP) depends on the number of integer and binary variables. Also, he proved that obtaining the optimal solutions for MILP models in a rational time is impossible when the number of variables is greater than 15. Besides, Mansini and Speranza [65] proved that solving the portfolio selection model with round lots is NP-hard. Therefore, a genetic algorithm (GA) is employed to solve the proposed models. The parameters of GA and NSGA-II are adjusted as follows: *POP*_*size*_: 100; crossover rate: 0.8; mutation rate: 0.4; maximum iteration: 500.

- **Portfolio selection using Model (1)**

**Fig 2:**
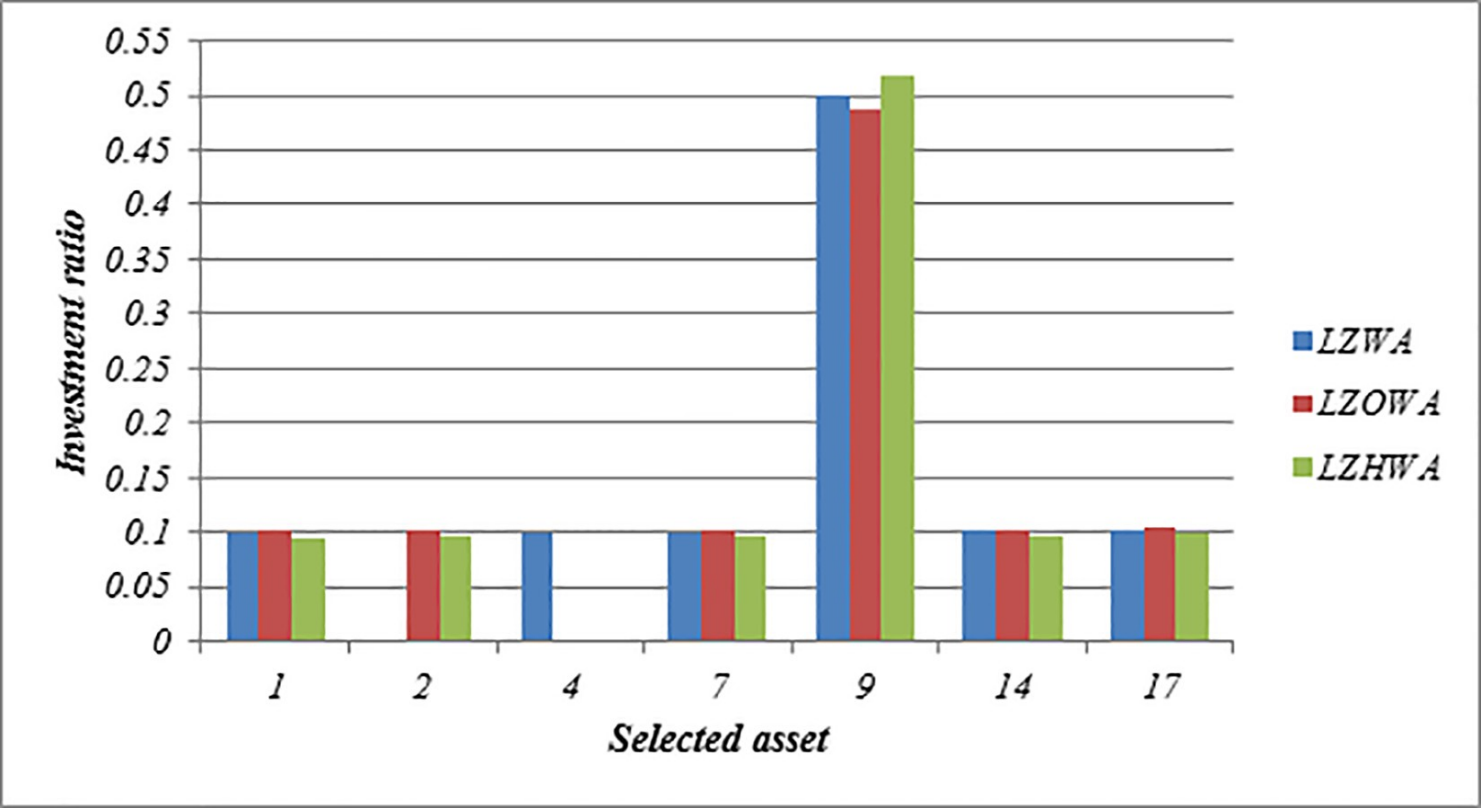
The selected assets and their investment ratio using Model (1).

**Fig 3:**
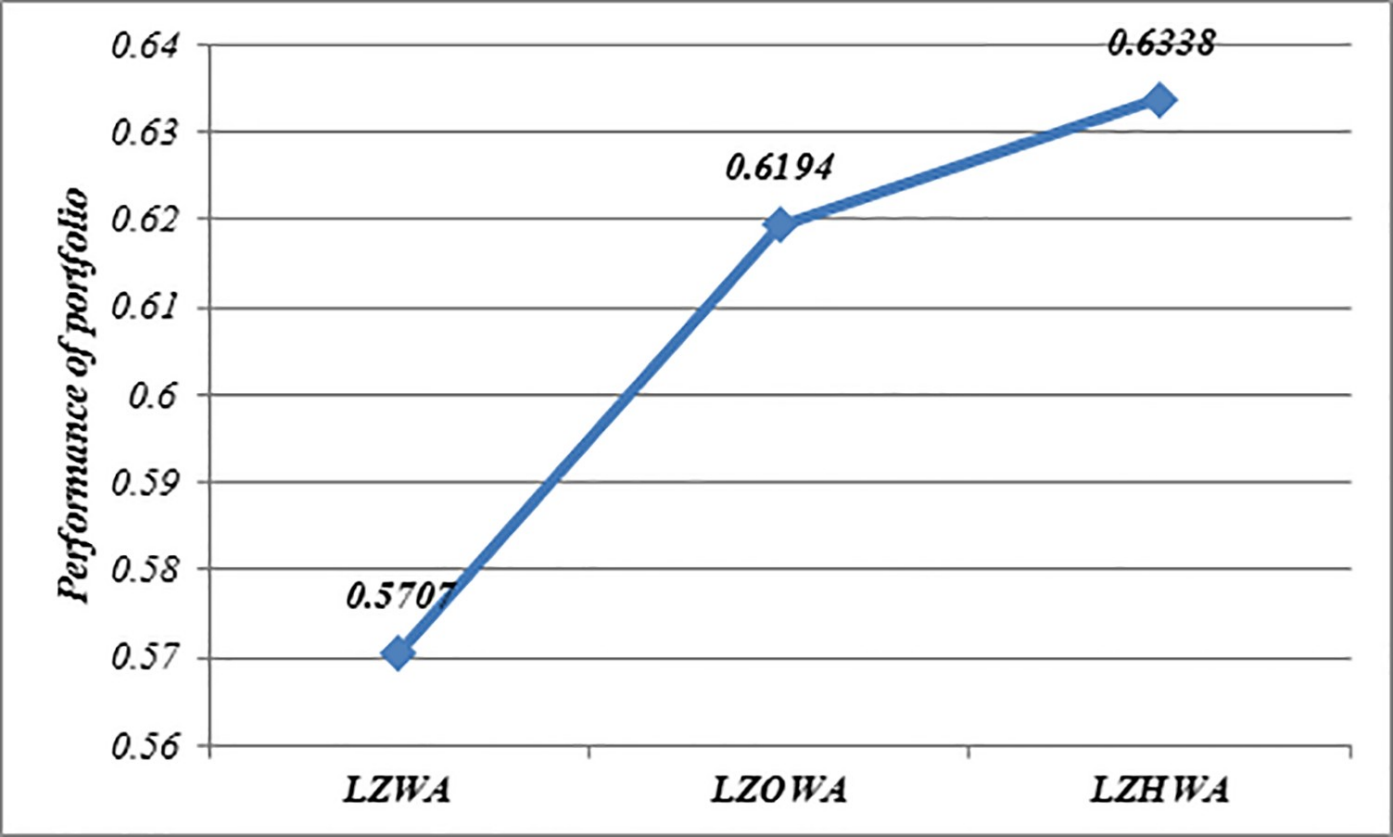
Comparison of the achievement level of goal resulted by Model (1) based on the proposed operators.

In this case, *h* = 6, *l*_*i*_ = 0.05 and *u*_*i*_ = 0.5 are used to build Model (1) based on LZWA, LZOWA, LZHWA operators. The selected portfolios are reported in Table 7. Fig 2 indicates that the performance of asset 9 is better than other assets under all proposed operators because this asset has a high score value in comparison with other assets. Moreover, it can be seen that the portfolios selected using Model (1) based on three proposed aggregation operators are nearly the same. Besides, Fig 3 shows that the achievement level of the goal becomes higher when LZHWA operator is applied to aggregate the evaluation information.

**Table 7:** The selected assets and their investment ratio in the portfolio by Model (1).

Operator	goal	Selected assets and their investment ratio						
LZWA								
0.5707		Asset ID	1	4	7	9	14	17
		Investment ratio	0.099	0.100	0.099	0.500	0.101	0.102
LZOWA								
0.6194		Asset ID	1	2	7	9	14	17
		Investment ratio	0.103	0.101	0.102	0.487	0.103	0.104
LZHWA								
0.6338		Asset ID	1	2	7	9	14	17
		Investment ratio	0.095	0.097	0.095	0.518	0.097	0.098

- **Portfolio selection using Model (2)**

**Fig 4:**
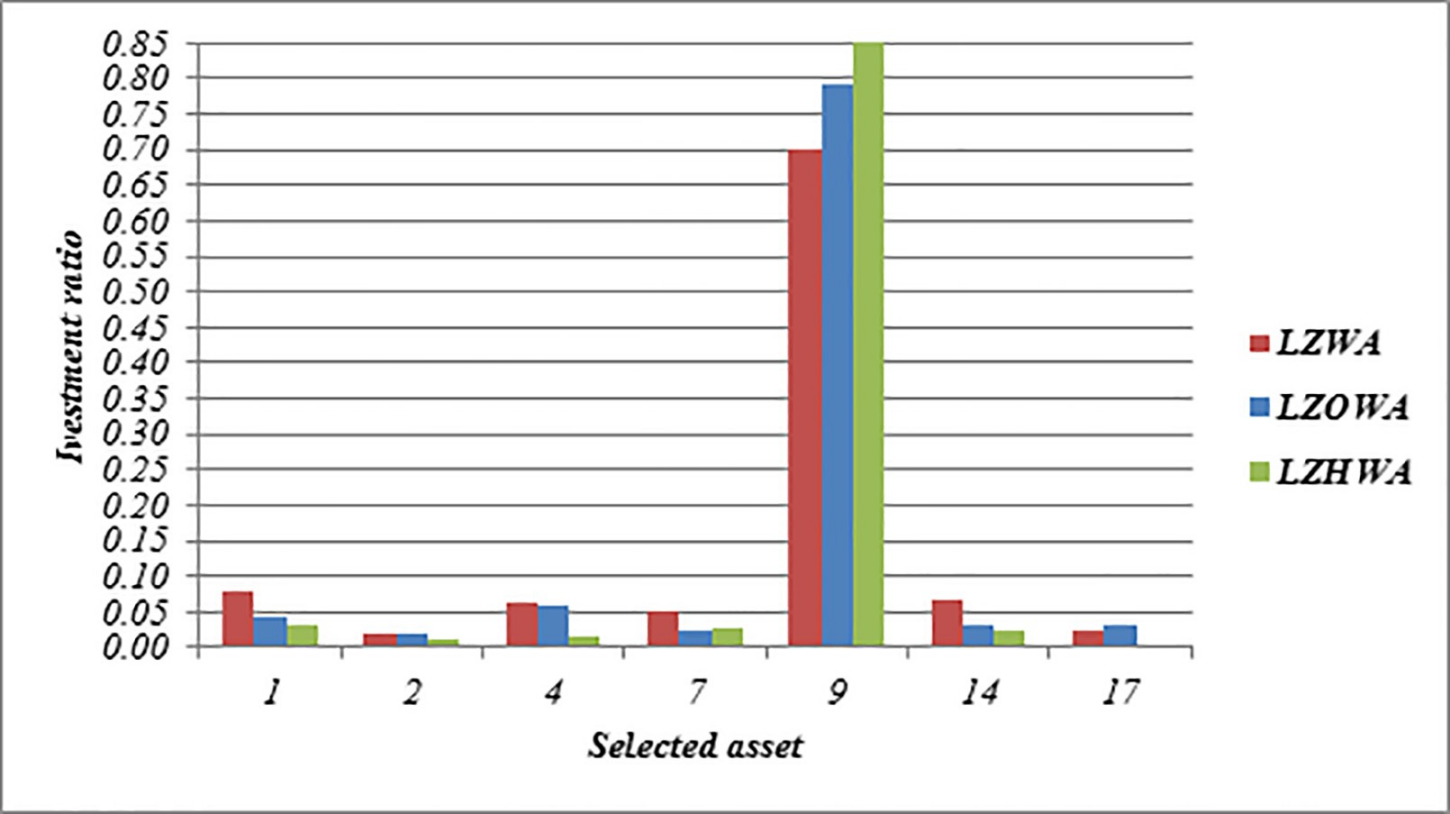
The selected assets and their investment ratio using Model (2).

**Fig 5:**
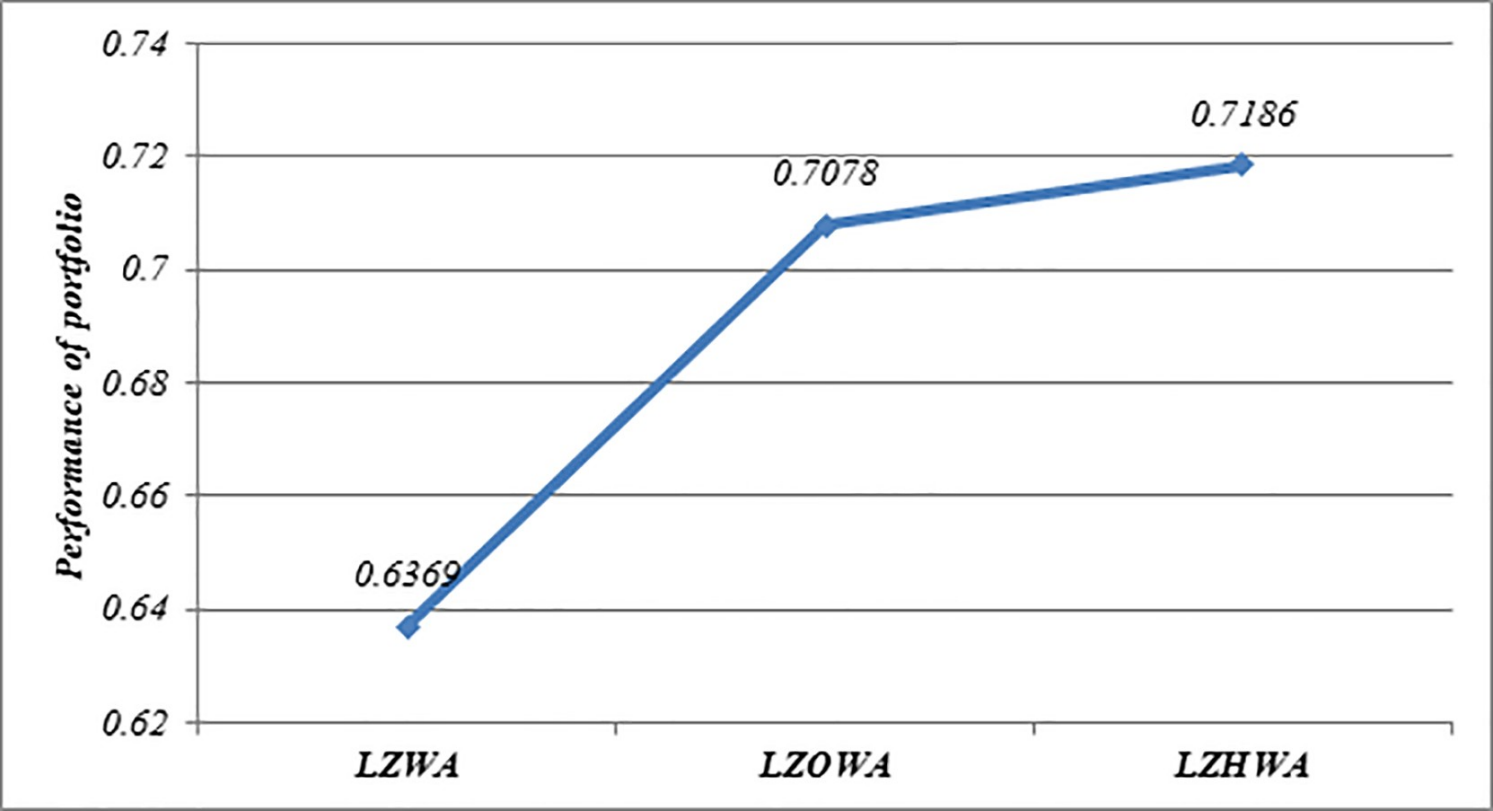
Comparison of the achievement level of goal resulted by Model (2) based on the proposed operators.

In this case, *δ* = 1.5 is applied to build Model (2) based on LZWA, LZOWA, LZHWA operators. The selected portfolios are listed in Table 8. Fig 4 indicates that the performance of asset 9 is better than other assets under all proposed operators because this asset has a high score value in comparison with other assets. Moreover, it can be found that the portfolios selected using Model (2) based on three proposed aggregation operators are nearly the same. Besides, Fig 5 shows that the achievement level of the goal becomes higher when LZHWA operator is applied to aggregate the evaluation information.

**Table 8:** The selected assets and their investment ratio in the portfolio by Model (2).

Operator	goal	Selected assets and their investment ratio							
LZWA									
0.6369		Asset ID	1	2	4	7	9	14	17
		Investment ratio	0.077	0.018	0.063	0.050	0.700	0.068	0.024
LZOWA									
0.7078		Asset ID	1	2	4	7	9	14	17
		Investment ratio	0.045	0.020	0.061	0.022	0.790	0.032	0.030
LZHWA									
0.7186		Asset ID	1	2	4	7	9	14	17
		Investment ratio	0.030	0.010	0.015	0.027	0.890	0.023	0.005

- **Portfolio selection using Model (3)**

**Fig 6:**
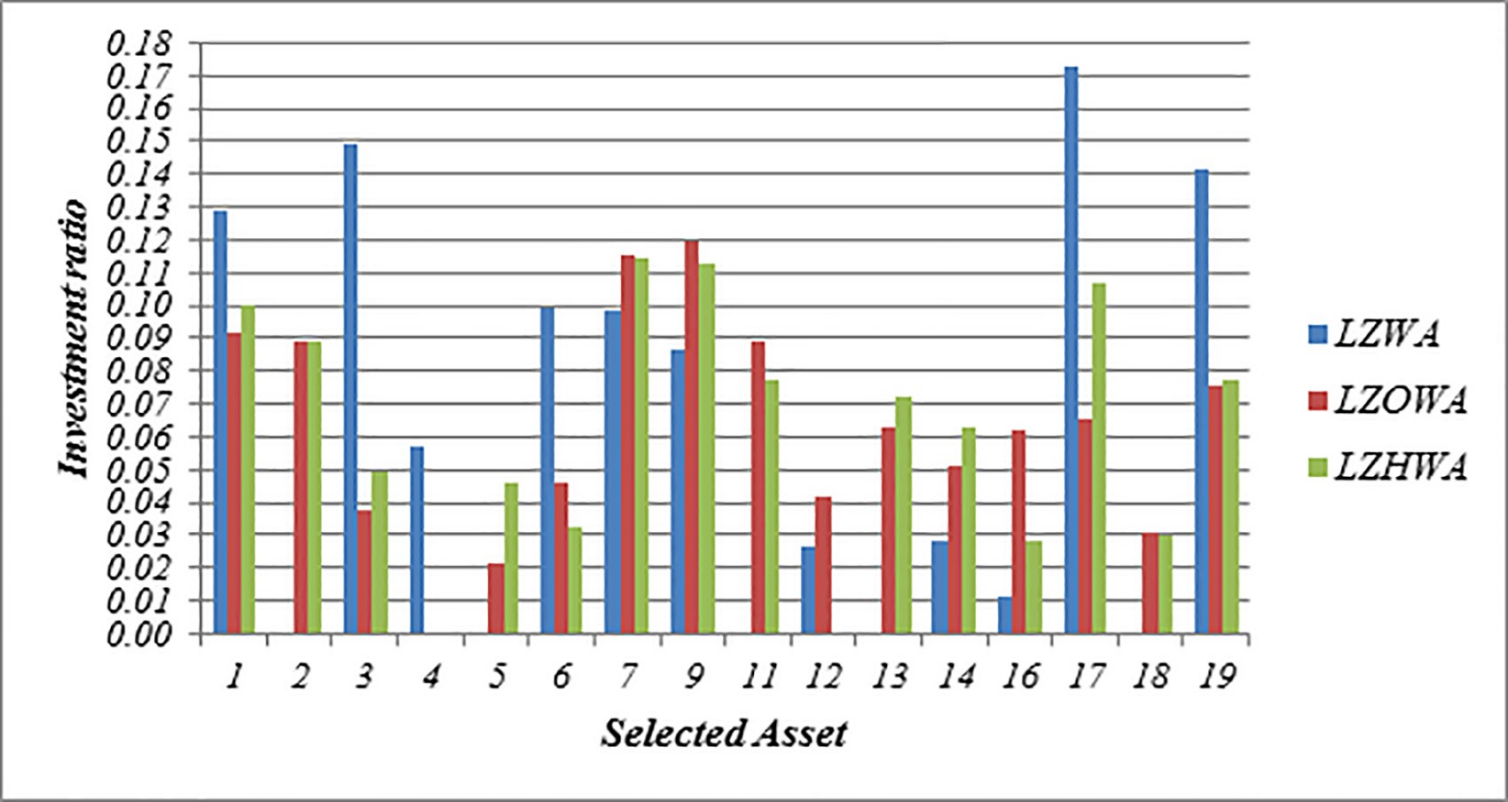
The selected assets and their investment ratio using Model (3).

**Fig 7:**
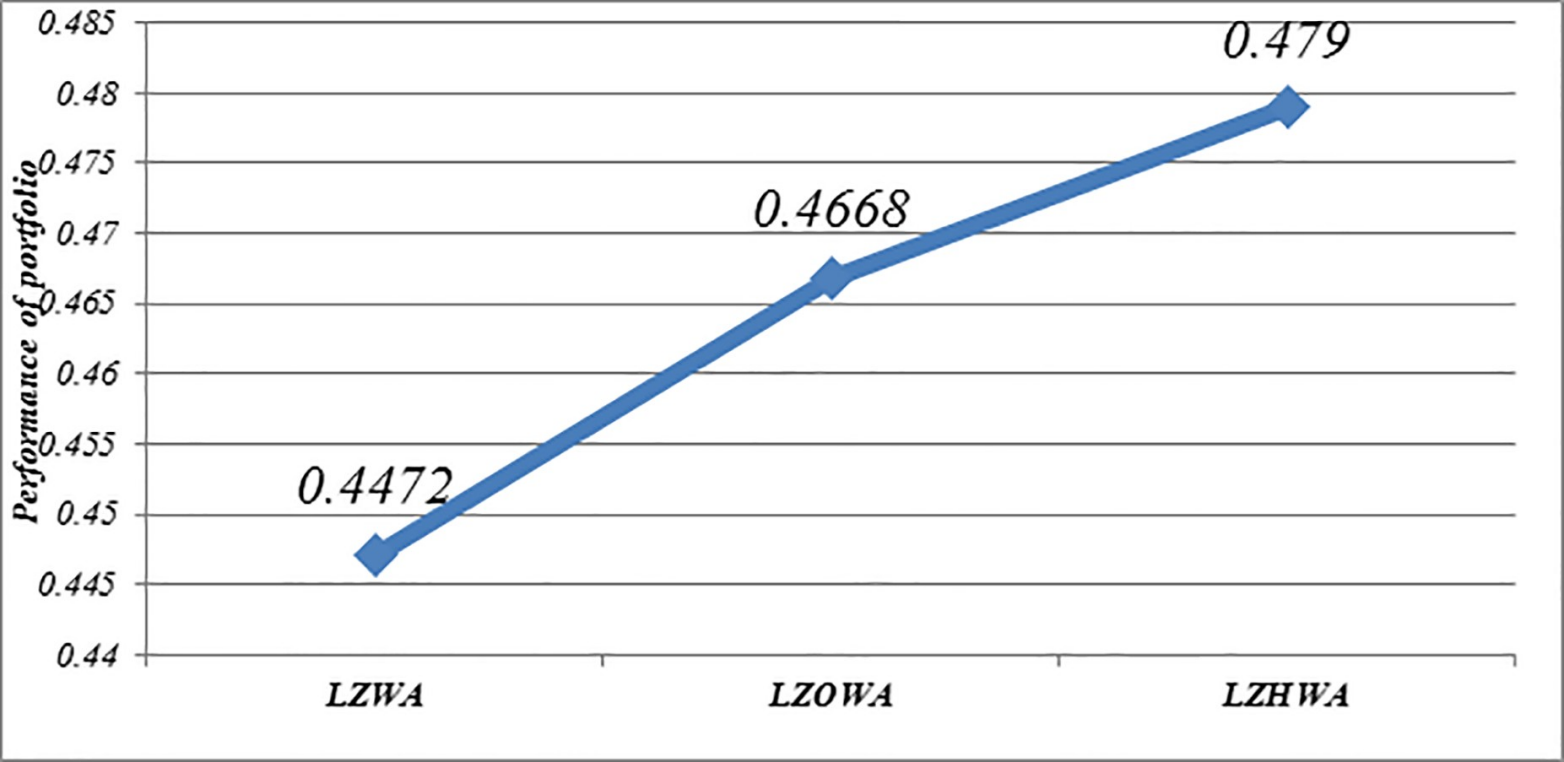
Comparison of the achievement level of goal resulted by Model (3) based on the proposed operators.

In this case, *δ* = 1.5 and *ϑ* = 0.15 is applied to build Model (2) based on LZWA, LZOWA, LZHWA operators. The selected portfolios are listed in Table 9. Fig 6 shows that the assets 7, 9 and 17 have better performance in comparison with other assets under all proposed operators due to the impact of reliability measure. Moreover, Model (3) determines the optimal asset allocation based on a trade-off between the score value and accuracy value. Also, it can be found that the portfolios selection using Model (3) based on three proposed aggregation operators are slightly different and it shows an advantage of our proposed aggregation operators. Besides, Fig 7 shows that the achievement level of the goal becomes higher when LZHWA operator is applied to aggregate the evaluation information.

**Table 9:** The selected assets and their investment ratio in the portfolio by Model (3).

Asset ID	LZWA	LZOWA	LZHWA	
1	0.129	0.092	0.100	
2	0	0.090	0.089	
3	0.149	0.037	0.049	
4	0.057	0	0	
5	0	0.022	0.046	
6	0.099	0.046	0.033	
7	0.099	0.115	0.115	
8	0	0	0	
9	0.087	0.119	0.113	
10	0	0	0	
11	0	0.089	0.077	
12	0.026	0.042	0	
13	0	0.063	0.072	
14	0.028	0.052	0.063	
15	0	0	0	
16	0.011	0.062	0.029	
17	0.173	0.065	0.107	
18	0	0.031	0.030	
19	0.141	0.076	0.077	
20	0	0	0	
The achievement level of goal	0.4472	0.4668	0.479	

### 5.4. Discussion and sensitivity analysis

It can be mentioned that the main feature of Model (3) emphasizes on the trade-off among two conflicting goals. This model can select the optimal portfolio by maximizing the score function, as a proxy of the expected return, along with the desired level of accuracy value as a risk constraint. In the following, the influence of critical parameters on Model (3) is analyzed.

In this case, sensitivity analysis is implemented by changing: (1) the minimum admissible level of accuracy value *ϑ* and (2) the preset entropy value *δ*. Tables 10–12 report the computational results.

**Table 10:** Portfolio selection by applying Model (3) based on LZWA operator.

Entropy level *δ*	1			1.5			2.000		
Accuracy level *ϑ*	0.1	0.15	0.2	0.1	0.15	0.2	0.1	0.15	0.2
Asset ID									
1	0.009	0.094	0.009	0.025	0.129	0.006	0.015	0.072	0.012
2	0.002	0	0.005	0	0	0.017	0.002	0	0.018
3	0.002	0.168	0.373	0.002	0.149	0.314	0.002	0.139	0.321
4	0.003	0.031	0.005	0.002	0.057	0.003	0.003	0.035	0.006
5	0.001	0	0	0.001	0	0	0.001	0	0
6	0.003	0.071	0	0.002	0.099	0.001	0.001	0.091	0
7	0	0.050	0.012	0.066	0.099	0.006	0.010	0.115	0.006
8	0.002	0	0.002	0.002	0	0.002	0.002	0	0.003
9	0.623	0.121	0.033	0.560	0.087	0.014	0.610	0.123	0.026
10	0.001	0	0	0.001	0	0	0.001	0	0.001
11	0.001	0	0.002	0.002	0	0.057	0.001	0.033	0.005
12	0.001	0	0	0	0.026	0	0	0.060	0
13	0.001	0	0.001	0.002	0	0.001	0	0	0.003
14	0.005	0.076	0.010	0.003	0.028	0.004	0.002	0.040	0.007
15	0.001	0	0.002	0.001	0	0.001	0.003	0	0.001
16	0.004	0.039	0.003	0.002	0.011	0.005	0.002	0.012	0.002
17	0.339	0.216	0.408	0.329	0.173	0.355	0.340	0.159	0.386
18	0	0	0	0.001	0	0	0.001	0	0
19	0.002	0.135	0.133	0.001	0.141	0.211	0.003	0.120	0.203
20	0.001	0	0.001	0	0	0	0.002	0	0.001
Achievement level of goal	0.5921	0.457	0.4174	0.5764	0.4472	0.4057	0.5606	0.4463	0.3923

**Table 11:** Portfolio selection by applying Model (3) based on LZOWA operator.

Entropy level *δ*	1			1.5			2.000		
Accuracy level *ϑ*	0.1	0.15	0.2	0.1	0.15	0.2	0.1	0.15	0.2
Asset ID									
1	0	0.083	0.038	0	0.070	0.013	0	0.090	0.023
2	0.234	0.108	0.066	0.211	0.069	0.094	0.083	0.083	0.047
3	0.007	0.068	0.111	0.001	0.061	0.110	0.015	0.064	0.118
4	0	0	0	0	0	0	0	0	0
5	0	0.045	0.087	0.001	0.024	0.098	0.001	0.019	0.113
6	0.001	0.038	0	0.001	0.043	0	0.001	0.032	0
7	0	0.102	0.053	0.001	0.114	0.060	0.001	0.111	0.026
8	0	0	0	0	0	0.001	0.001	0	0
9	0.518	0.100	0.026	0.604	0.110	0.031	0.694	0.125	0.041
10	0	0	0	0.001	0	0	0.001	0	0
11	0.006	0.065	0.126	0.002	0.069	0.109	0.038	0.089	0.096
12	0	0.022	0.097	0.001	0.033	0.101	0.001	0.035	0.109
13	0.038	0.096	0.143	0.127	0.079	0.141	0.111	0.066	0.136
14	0	0.065	0.031	0	0.083	0.017	0	0.043	0.044
15	0	0	0	0	0	0	0	0	0
16	0	0.068	0.013	0	0.062	0.050	0	0.063	0.059
17	0.163	0.087	0.065	0.046	0.086	0.034	0.051	0.100	0.048
18	0	0.006	0	0.001	0.036	0	0.002	0.035	0.005
19	0.031	0.048	0.143	0.003	0.062	0.141	0.001	0.047	0.136
20	0	0	0	0	0	0	0	0	0
Achievement level of goal	0.6024	0.4663	0.3807	0.623	0.4789	0.3978	0.6439	0.4899	0.4127

**Table 12:** Portfolio selection by applying Model (3) based on LZHWA operator.

Entropy level *δ*	1			1.5			2		
Accuracy level *ϑ*	0.1	0.15	0.2	0.1	0.15	0.2	0.1	0.15	0.2
Asset ID									
1	0.001	0.111	0.043	0.001	0.089	0.038	0	0.097	0.049
2	0.124	0.082	0.054	0.015	0.102	0.043	0.272	0.103	0.055
3	0.003	0.055	0.117	0.002	0.059	0.120	0.001	0.057	0.109
4	0	0	0	0	0	0	0	0	0
5	0	0.045	0.095	0.002	0.018	0.053	0.001	0.049	0.098
6	0.001	0.014	0	0.001	0	0	0.002	0	0
7	0.096	0.109	0.060	0.077	0.108	0.058	0.104	0.117	0.030
8	0	0	0	0	0	0	0	0	0.037
9	0.613	0.107	0.062	0.697	0.138	0.039	0.532	0.136	0.047
10	0.001	0.000	0	0.001	0	0	0	0	0
11	0.003	0.070	0.116	0.001	0.062	0.099	0.001	0.082	0.120
12	0.001	0.023	0.083	0.001	0.028	0.088	0.001	0.030	0.081
13	0.102	0.078	0.132	0.200	0.120	0.146	0.081	0.094	0.128
14	0	0.059	0.052	0	0.019	0.026	0	0.067	0.015
15	0	0	0	0	0	0	0	0	0
16	0	0.067	0.016	0	0.056	0.025	0	0.045	0.047
17	0	0.079	0.034	0	0.098	0.079	0	0.072	0.041
18	0	0.015	0.024	0.001	0	0.048	0.001	0.001	0.044
19	0.052	0.085	0.110	0	0.102	0.139	0.003	0.049	0.097
20	0	0	0	0	0	0	0	0	0
Achievement level of goal	0.6162	0.4655	0.3905	0.6294	0.4891	0.4095	0.6474	0.5035	0.4286

**Fig 8:**
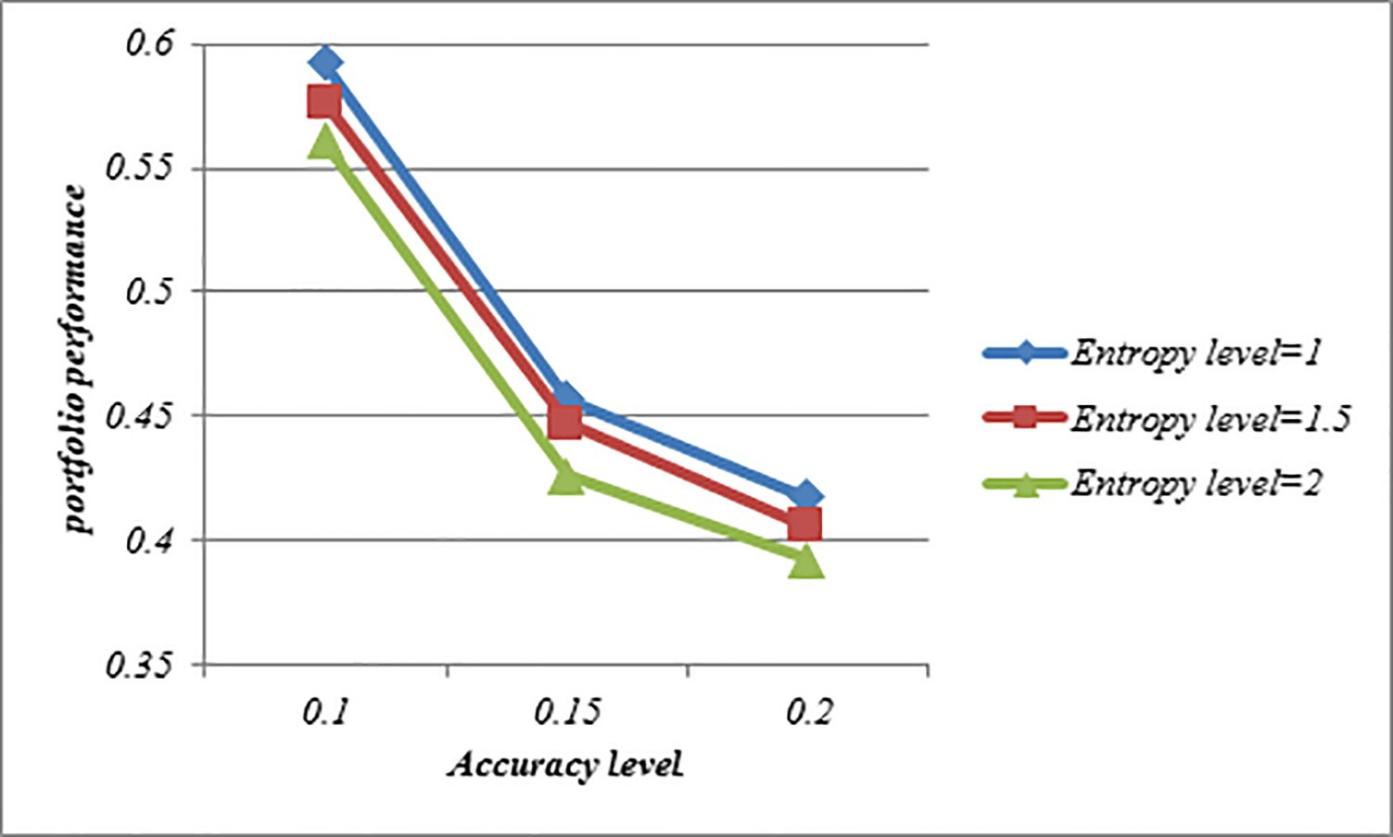
Comparison of the achievement level of goal in Model (3) based on LZWA operator.

**Fig 9:**
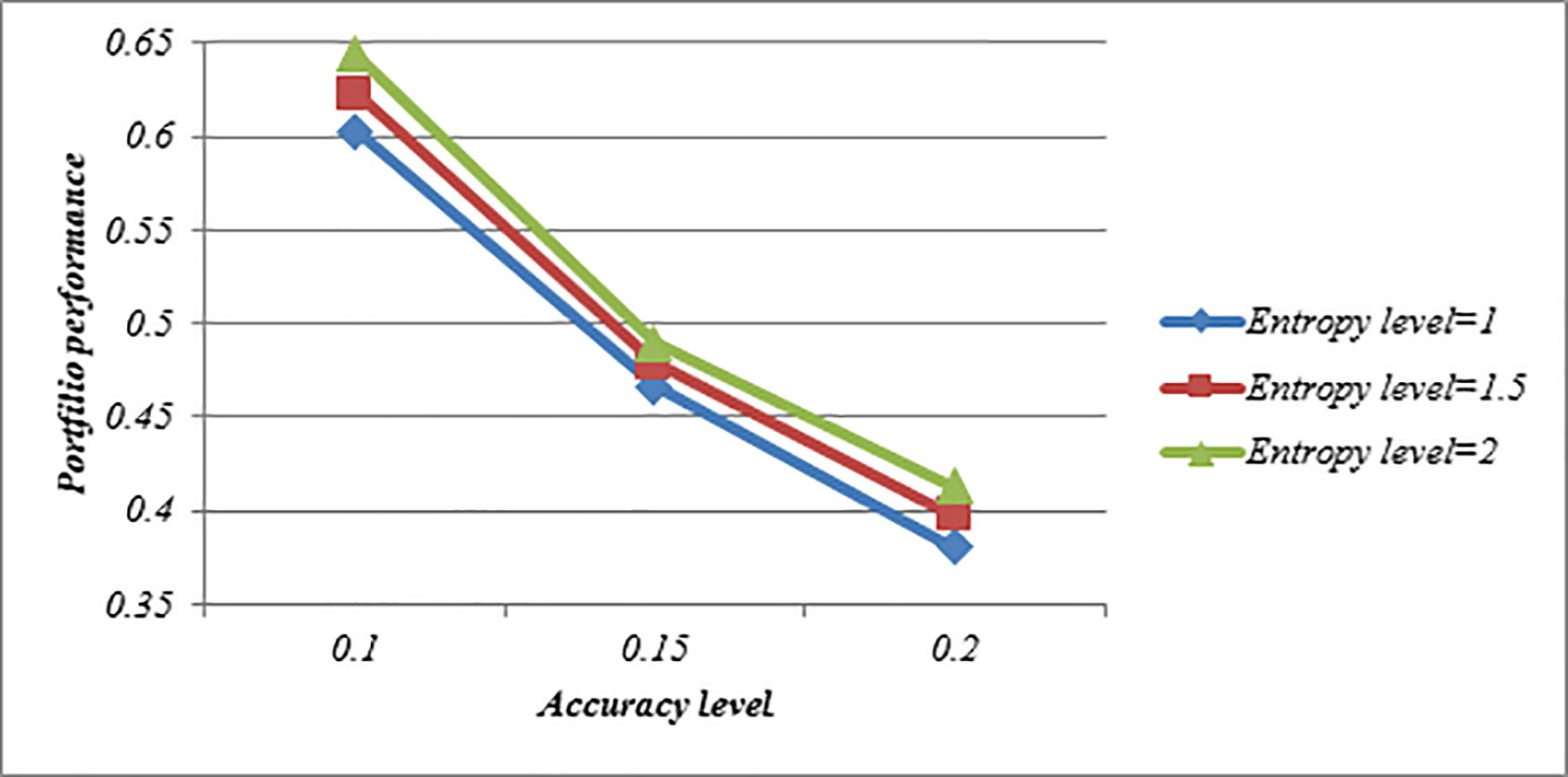
Comparison of the achievement level of goal in Model (3) based on LZOWA operator.

**Fig 10:**
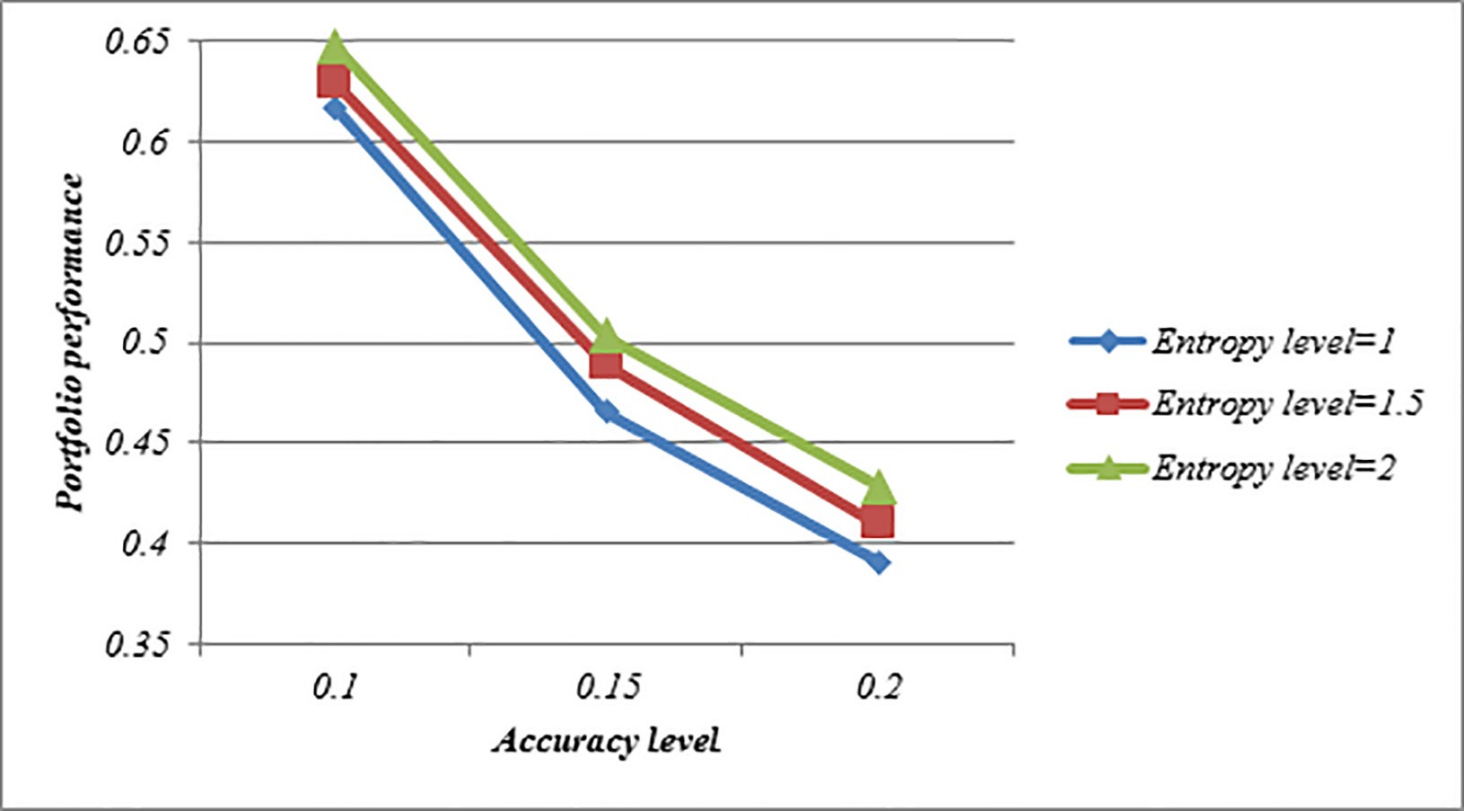
Comparison of the achievement level of goal in Model (3) based on LZHWA operator.

Figs 8–10 indicate that the achievement level of goal becomes lower under all situations when *ϑ*-value is increased. This feature shows that Model (3) can create a comprehensive trade-off between the score value and the accuracy value in the portfolio. Moreover, with increasing *ϑ*-value, the investment ratio of some assets such as 3, 11, 13 and 19 become higher, and vice versa, the investment ratio of some assets such as 2, 9, 14 and 17 become lower which can be seen in Tables 10–12. Therefore, it can be found that Model (3) is more proper for risky investors as they are intending to obtain the maximum return along with a limited risk level. Moreover, it can be noted that the determination of ϑ-value may depend on the mentality of the investor. A risk averter investor can select higher ϑ-value, but a risk seeker investor can select lower ϑ-value. Therefore, this issue can provide additional useful information to help the investors efficiently make fruitful decisions. Also, Figs 8–10 indicate that the achievement level of goal becomes higher under all situations when the entropy level (*δ*-value) increases. Thus, the investors can construct more portfolios by changing the predefined entropy value in the Model (3) according to their preferences.

### 5.5. Comparative analysis

#### 5.5.1. Comparing with fuzzy weighted averaging (FWA), fuzzy ordered weighted averaging (FOWA) and fuzzy hybrid weighted averaging (FHWA) [55]

FWA, FOWA and FHWA operators were presented by Xu and Da [55] and these operators only can handle fuzzy information or linguistic information. However, fuzzy numbers can’t capture expert’s reliability in describing qualitative information. Experts always express their opinions in terms of various possible linguistic terms with different reliability. The Linguistic Z-numbers covers their requirements. But FWA, FOWA and FHWA operators can’t handle decision-making problems under the linguistic Z-number environment. Since linguistic Z-numbers are quite different from fuzzy numbers, we develop these operators under the linguistic Z-number environment using the corresponding operational rules.

#### 5.5.2. Comparing with Zhou et al.’s method [66]

To investigate the practicability and validity of the proposed qualitative approach, the proposed method is compared with Zhou et al.’s method [66]. Zhou et al. [66] proposed a qualitative portfolio selection approach based on aggregation operators under the hesitant fuzzy environment. Thus, the linguistic Z-number data can be rewritten as hesitant fuzzy linguistic information. the hesitant fuzzy linguistic term sets consider many possible linguistic terms. However, they do not contain the reliability measure of these linguistic terms. Therefore, it is assumed that all possible linguistic terms have the same reliability values under the hesitant fuzzy linguistic environment. Based on the linguistic Z-number information shown in Table 1, the linguistic Z-number decision matrix can be rewritten as the hesitant fuzzy linguistic decision matrix. For example, the linguistic Z-valuation $\left(x_{11},s_{6},s_{3}^{\prime }\right)$, which evaluates the performance of asset 1 concerning the first criterion, can be rewritten as the hesitant fuzzy linguistic value <*x*_11_,*s*_6_>. Then, the linguistic term *s*_6_ can be transformed to triangular fuzzy number based on Herrera et al.’s method [67]. Therefore, the hesitant fuzzy linguistic value <*x*_11_,*s*_6_> can be rewritten as hesitant fuzzy value <*x*_11_,{0.49,0.67,0.84}>. Zhou et al. [66] used the max-score value rule to formulate his proposed approach. Therefore, we compare the results of the Model (1) with the results of Zhou’s model [66].

The Zhou et al.’s model with consideration of cardinality constraint is applied to select the optimal portfolio under hesitant fuzzy environment. Table 13 shows the assigned assets and their investment ratios resulted in Zhou et al.’s model. Now, we compare the results obtained by Model (1) in Table 7 with the results obtained in Zhou et al.’s model in Table 13.

**Table 13:** The selected portfolios obtained by the Zhou’s model based on the max-score rule.

Asset ID	Investment ratio	Asset ID	Investment ratio	Asset ID	Investment ratio
1	0.12	3	0.0994	7	0.165
2	0.119	9	0.333	17	0.165

From Tables 7 and 13, it can be seen that the portfolio selected by our proposed model is almost different from the portfolio selected in Zhou et al.’s model. This difference shows an advantage of our proposed approach which captures the reliability of evaluation information. The portfolio performance obtained in Zhou et al.’s model based on the max-score rule is equal to 0.412. Thus, the portfolio performances in the proposed model are better than the portfolio performance in Zhou et al.’s model. Moreover, with a more accurate investigation of the results obtained by our proposed model and shown in Table 7, we find that the proposed model completely considers the requirement of reliability. For example, some assets like asset 3 are not allocated to the portfolio obtained in Model (1) because experts have devoted lower values of reliability to their corresponding assessment data. Instead, these assets are selected in Zhou’s models because these models do not consider experts’ reliability. In other words, the proposed model can select the optimal assets by considering different criteria when experts are sure about their assessments. Therefore, we can indicate that the proposed approaches are superior and more general due to considering the reliability of the information.

### 5.6. Managerial results

As discussed above, the proposed qualitative approach contains the incorporation of aggregation techniques with portfolio selection problems under the linguistic Z-number environment. The proposed models are proposed to allocate the optimal assets. Model (1) and Model (2) are more suitable for general investors as they want to obtain the maximum return. Finally, Model (3) is suitable for risky investors who intend to attain the maximum return along with a desired accuracy level. Furthermore, the diversification of portfolios is guaranteed by considering the predefined number of assets in Model (1) and the entropy level in Model (2) and Model (3).

Thus, the advantages of our proposed approach are as follows:

1. Our proposed approach is more comprehensive in comparison with the traditional approaches because we use the linguistic Z-numbers in the assessment information representing. The linguistic Z-numbers not only reduce the loss of information but also capture the possibilistic and probabilistic constraints, simultaneously. So, they are more suitable for evaluating the information in financial markets.
2. Our proposed aggregation techniques can rank all input arguments in the decision-making environment. Although there are some operators to aggregate the assessment information under the fuzzy environment, they are unable to solve decision-making problems under linguistic Z-number environment. So, our proposed aggregation operators such as LZWA operator, LZOWA operator, and LZHWA operator are more general and more flexible to fuse the evaluation data. Our proposed aggregation operators not only rank all input arguments but also consider the reliability of the information.
3. We apply the max-score rule and the score-accuracy trade-off rule and propose three qualitative models to select the optimal portfolio under the linguistic Z-number environment. Thus, our proposed models are suitable for both general investors and the risky investors. Moreover, our proposed models not only can construct more diversified portfolios according to investor’s preferences but also they can test the trade-off between the score value and accuracy value under various situations.

## 6. Conclusion

To select the optimal portfolio when the assessment information is qualitative and linguistic, this study has proposed a holistic multi-stage methodology the under linguistic Z-number environment with considering both investor’s preferences and expert’s reliability. The main stages of the developed approach are as follows: (1) aggregating the linguistic Z-number data using WA, OWA and HMM operators, and developing three new aggregation operators (2) computing the score values and the accuracy values for each alternative (3) formulating three qualitative portfolio optimization models to assign the optimal assets according to different preferences of investor. Moreover, three qualitative portfolio selection models have been developed based on the max-score rule and the score-accuracy trade-off rule to allocate assets based on a real case. The results show that the proposed framework can generate more diversified portfolios based on a trade-off between the score value and accuracy value. Moreover, the proposed framework not only can capture investor’s preferences but also can consider the expert’s reliability in the investment processes. Besides, by considering various levels of the portfolio risk, the proposed qualitative approach has been extended to distinguish the risk averter and risk seeker investors. The comparison results show that the portfolio performance of the proposed approach has been increased 20 percent in comparison with the other approach.

In this study, the proposed approach applies opinions of one expert to evaluate the performance of assets with respect to different criteria under linguistic Z-number environment. However, the proposed method can be extended to the case which more than one expert’s opinions is considered. This problem is referred as the group portfolio selection under linguistic Z-number environment. When this approach is employed to allocate the optimal assets based on the linguistic Z-number evaluation data provided by *t* experts or DMs, *t* linguistic Z-number matrices are acquired and the linguistic Z-number aggregation operators can be used to combine them into a collective linguistic Z-number matrix. The rest of modelling processes is the same as the proposed method in this paper. This issue remains a subject of future study. For more information, readers are referred to the work by Wang et al. [68] and Chen et al. [69]. Moreover, in this paper, we have considered aggregation operators for linguistic Z-number environment. However, source of uncertainty may be from different types. Therefore, the proposed aggregation operators of this paper can be extended to support heterogenous interrelationships among attributes. Interested researchers can read the work by Chen et al. [70]. Ultimately, this study uses Xu’s method [63] to determine the weights of criteria. However, the weight information in the real decision-making processes may be incomplete. Thus, to determine the incomplete weights of criteria in the proposed approach, other methods such as Chen et al.’s method [71], Kim and Ahn’s method [72] and Zhang et al.’s method [73] can be applied. This remains as a subject for future study.
